# ALK2 inhibitors display beneficial effects in preclinical models of *ACVR1* mutant diffuse intrinsic pontine glioma

**DOI:** 10.1038/s42003-019-0420-8

**Published:** 2019-05-09

**Authors:** Diana Carvalho, Kathryn R. Taylor, Nagore Gene Olaciregui, Valeria Molinari, Matthew Clarke, Alan Mackay, Ruth Ruddle, Alan Henley, Melanie Valenti, Angela Hayes, Alexis De Haven Brandon, Suzanne A. Eccles, Florence Raynaud, Aicha Boudhar, Michelle Monje, Sergey Popov, Andrew S. Moore, Jaume Mora, Ofelia Cruz, Mara Vinci, Paul E. Brennan, Alex N. Bullock, Angel Montero Carcaboso, Chris Jones

**Affiliations:** 1 Divisions of Molecular Pathology, The Institute of Cancer Research, London, SM2 5NG UK; 2 Division of Cancer Therapeutics, The Institute of Cancer Research, London, SM2 5NG UK; 3 Stanford University School of Medicine, Stanford, 94305 CA USA; 4 Institut de Recerca Sant Joan de Deu, Barcelona, 08950 Esplugues de Llobregat Spain; 5 Structural Genomics Consortium, University of Oxford, Oxford, OX3 7DQ UK; 6 Nuffield Department of Medicine, Target Discovery Institute, University of Oxford, Oxford, OX3 7FZ UK; 7 Department of Cellular Pathology, University Hospital of Wales, Cardiff, CF14 4XW UK; 8 Diamantina Institute and Child Health Research Centre, The University of Queensland, Brisbane, QLD 4101 Australia; 9 Oncology Service, Queensland Children’s Hospital, Brisbane, QLD 4029 Australia; 10 Bambino Gesù Children’s Hospital, Rome, 00165 Roma RM Italy

**Keywords:** Target validation, CNS cancer, Paediatric cancer

## Abstract

Diffuse intrinsic pontine glioma (DIPG) is a lethal childhood brainstem tumour, with a quarter of patients harbouring somatic mutations in *ACVR1*, encoding the serine/threonine kinase ALK2. Despite being an amenable drug target, little has been done to-date to systematically evaluate the role of *ACVR1* in DIPG, nor to screen currently available inhibitors in patient-derived tumour models. Here we show the dependence of DIPG cells on the mutant receptor, and the preclinical efficacy of two distinct chemotypes of ALK2 inhibitor in vitro and in vivo. We demonstrate the pyrazolo[1,5-a]pyrimidine LDN-193189 and the pyridine LDN-214117 to be orally bioavailable and well-tolerated, with good brain penetration. Treatment of immunodeprived mice bearing orthotopic xenografts of H3.3K27M, *ACVR1*R206H mutant HSJD-DIPG-007 cells with 25 mg/kg LDN-193189 or LDN-214117 for 28 days extended survival compared with vehicle controls. Development of ALK2 inhibitors with improved potency, selectivity and advantageous pharmacokinetic properties may play an important role in therapy for DIPG patients.

## Abstract (web summary)

Diana Carvalho, Kathryn R. Taylor et al. show that ALK2 inhibitors induce apoptosis of ACVR1 mutant diffuse intrinsic pontine glioma cells in vitro. These compounds also increase the survival of mice carrying ACVR1 mutant brainstem xenografts, suggesting ALK2 as a potential pharmacological target against this lethal cancer.

## Introduction

Diffuse intrinsic pontine glioma (DIPG) is an incurable infiltrating glioma of the brainstem in children, with a median overall survival of 9–12 months^1–3^. The mainstays of chemotherapy in histologically similar tumours of other anatomical brain regions are ineffective, in part due to the absence of factors which predict efficacy, such as *MGMT* promoter methylation and response to temozolomide^4^. The uniqueness of the underlying biology of DIPG is most readily demonstrated by the high prevalence (>80%) of lysine-to-methionine substitutions at position 27 (K27M) in genes encoding histone H3.1 (*HIST1H3B, HIST1H3C*) and H3.3 variants (*H3F3A*)^5,6^. To date, radiotherapy provides the only therapeutic response, although >90% children suffer a relapse and die from their disease within 2 years^1,3,7^.

Recent collaborative molecular sequencing initiatives have defined the genomic landscape of DIPG. We and others identified recurrent somatic activating mutations in the gene *ACVR1* in ~25% of DIPG patients^8–11^. Whilst H3.3 K27M mutations are also present in other midline regions such as the thalamus, spine and cerebellum (diffuse midline glioma with H3K27M mutation in the 2016 WHO classification schema^12^), *ACVR1* mutations are associated with H3.1K27M substitutions, and appear restricted to DIPG^13^. In keeping with the clinicopathological differences between H3.3 and H3.1 K27M mutant subgroups^14^, *ACVR1* mutations have been reported at a younger age of diagnosis and with longer overall survival in children with DIPG^8–11^. Although apparently not found in any other human cancer, these variants are found in the germline of patients with the congenital malformation syndrome fibrodysplasia ossificans progressiva (FOP), in which soft tissue is remodelled to bone in response to (often trauma-related) inflammation^15^. In the brain, activin A—ACVR1 signalling is involved in the process of myelination^16–18^, an intriguing association given the putative oligodendroglial precursor origins of DIPG^18–20^.

*ACVR1* encodes the receptor serine/threonine kinase ALK2, and in models of FOP, it has recently been reported that the characteristic mutations confer an aberrant sensitivity to the ligand activin A, produced as part of the inflammatory response, rather than the canonical BMPs^21^. This results in increased pathway activation and cell signalling via a canonical phosphorylated SMAD1/5/8-SMAD4 pathway to drive expression of target genes including *ID1/2/3*, *SNAIL* and *HEY1*^22^. Inhibition of this response provides the major impetus of preclinical development and clinical trials in FOP.

The first small molecule inhibitor of ALK2 was dorsomorphin, identified through a classical BMP ventralization assay in zebrafish embryos^23^. The pyrazolo[1,5-a]-pyrimidine scaffold of dorsomorphin provided the basis of further compounds demonstrated to target ALK2 and the related BMP receptors ALK3 and ALK6, without affecting the type I TGF-beta receptor ALK5^24^. Continued improvements in selectivity have been reported in a newer series based instead on a pyridine scaffold, the prototype of which is K02288^25^.

In the present study, we demonstrate *ACVR1* mutations confer abnormal ligand responsiveness to activin A in DIPG cells, with spatiotemporal expression of activin A in neurodevelopment correlating with tumour origins. Screening mutant and wild-type DIPG cultures with a range of pyrazolo[1,5-a]pyrimidine- and pyridine-based ALK2 inhibitors demonstrated differential effects on cell viability, recapitulating genetic knockdown with shRNA, and prolongation of survival in orthotopic patient-derived xenograft models of DIPG.

## Results

### Clinical and molecular correlates of *ACVR1* mutant DIPGs

**Fig. 1:**
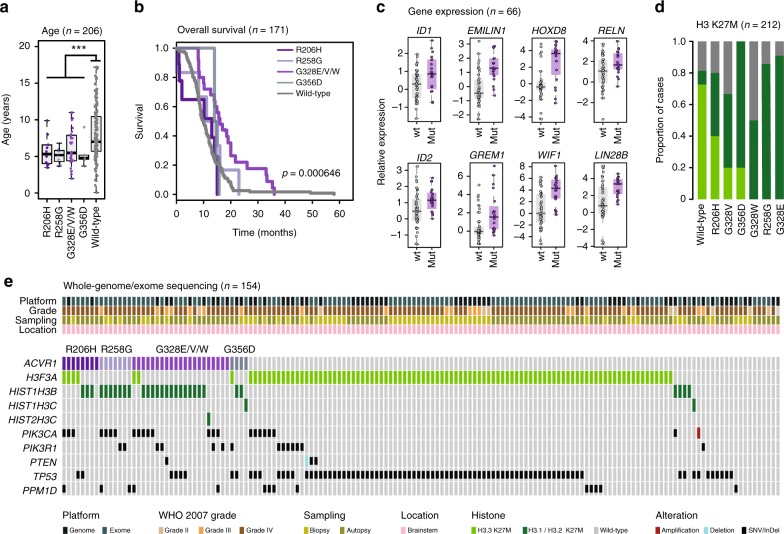
Somatic *ACVR1* mutations in DIPG. **a** Boxplot showing age at diagnosis of DIPG cases, separated by *ACVR1* variant (*n* = 206). The lower and upper limits of the boxes represent the first and third quartiles, and the whiskers 1.5x the interquartile range. *** adjusted *p* < 0.0001 for all pairwise comparisons, *t*-test. **b** Kaplan–Meier plot of overall survival of cases separated by *ACVR1* variant, *p*-value calculated by the log-rank test (*n* = 171). **c** Boxplots representing gene expression differences between *ACVR1* mutant (purple) vs wild-type cases (grey) in an integrated gene expression dataset (*n* = 66). **d** Stacked barplot of distribution of H3 K27M mutations separated by *ACVR1* variants. Dark green = H3.1 K27M, light green = H3.3 K27M, grey = wild-type (*n* = 212). **e** Oncoprint representation of an integrated annotation of somatic mutations and DNA copy number changes for selected genes in DIPG (*n* = 154). Samples are arranged in columns with genes labelled along rows. Clinicopathological and molecular annotations are provided as bars according to the included key

To assess the differences between distinct somatic variants, we re-examined data from a genomics meta-analysis comprising 212 DIPG cases for which *ACVR1* status was available^14^. We identified 50/212 (23.6%) cases with *ACVR1* mutation, and found no differences in age at diagnosis between R206H (*n* = 10, median = 5.35 years), R258G (*n* = 7, median = 5.2 years), G328E/V/W (*n* = 28, median = 5.5 years) and G356D (*n* = 5, 4.8 years) variants (*p* = 0.6957, ANOVA), though together, mutant cases were significantly younger than *ACVR1* wild-type DIPG (5.25 vs 7.0 years, *p* < 0.0001, *t*-test) (Fig. 1a). The presence of any *ACVR1* mutation conferred a longer overall survival (*p* = 0.00291, log-rank test), however this benefit was largely restricted to amino acid substitutions at the G328 residue (median survival = 16.0 months; *p* = 0.000646, log-rank test), with other mutations showing no significant differences in clinical outcome compared with *ACVR1* wild-type patients (wild-type median survival = 10.0 months; R206H = 13.0 months; R258G = 13.1 months; G356D = 14.3 months) (Fig. 1b). Of eight long-term survivors (>24.0 months), five were *ACVR1* mutant (62.5%), all of which were G328E/V/W; ranking DIPG patients by their overall survival, 10/21 (47.6%) of the top 10% survivors (>19.0 months) were *ACVR1* mutant, all but one G328E/V/W.

Gene expression data were available across three independent platforms for 66 patients (Supplementary Fig. 1a), with an integrated analysis identifying 244 differentially expressed genes between *ACVR1* mutant and wild-type DIPGs (adjusted *p* < 0.05, Mann–Whitney *U* test) (Supplementary Table 1). *ACVR1* mutation was significantly associated with upregulation of known BMP/TGFβ target genes such as *ID1* (*p* = 0.0261) and *ID2* (*p* = 0.0101), as well as other regulators (*EMILIN1*, *p* = 0.00000218) and antagonists (*GREM1*, *p* = 0.00187), in addition to genes associated with neurogenesis (*WIF1*, *p* = 0.0000556), neuronal migration (*RELN*, *p* = 0.0292), differentiation (*HOXD8*, *p* = 0.000132) and pluripotency (*LIN28B*, *p* = 0.000714) (Fig. 1c). By gene set enrichment analysis, there was co-ordinated regulation of expression signatures associated with signalling through HIF1, TGFβ, WNT, STAT3 and TP53 pathways (Supplementary Fig. 1b).

There was an expected strong association of *ACVR1* mutation with H3.1K27M (36/50 vs 8/50 H3.3 K27M and 6/50 H3 wild-type; *p* < 0.0001, Fisher's exact test), however, the distribution of histone mutations differed with distinct *ACVR1* variants. Notably, R206H mutant tumours contained equal proportions of H3.3 and H3.1K27M (Fig. 1d). DIPGs with more extensive genome or exome sequencing data (*n* = 154) allowed us to investigate additional co-segregating mutations, and identified a significant enrichment of PI3-kinase pathway alterations (*PIK3CA*, *PIK3R1* and *PTEN*) in *ACVR1* mutant compared with wild-type tumours (22/40, 55% vs 18/114, 15.8%; *p* < 0.0001), whilst conversely the TP53 pathway was targeted by mutations in *TP53* and *PPM1D* significantly more commonly in *ACVR1* wild-type cases (13/40, 32.5% vs 87/114, 76.3%; *p* < 0.0001, Fisher’s exact test) (Fig. 1e).

Taking the *ACVR1* mutations separately, tumours with R258G trended towards fewer copy number aberrations (*p* = 0.0658, *t*-test), whilst R206H had significantly fewer somatic mutations (0.0257, *t*-test) (Supplementary Fig. 1c). There was enrichment for *PIK3CA* mutations in R258G tumours, and *PIK3R1* in G356D (log2 odds ratios >2.5), however this failed to reach statistical significance. Screening all somatic mutations highlighted previously unreported co-segregation of *ACVR1* G328E/V/W with variants in the cyclin-dependent kinase inhibitor *CDKN2C* (*p* = 0.0113, Fisher's exact test) and the lysine demethylase *KDM6B* (*p* = 0.0238, Fisher's exact test) (Supplementary Fig. 1d). Gene expression differences between the various *ACVR1* mutations revealed an upregulation of genes involved in oligodendrocyte differentiation in G328E/V/W mutant tumours compared to R206H and G356D, including *MOG* (myelin oligodendrocyte glycoprotein), *MAG* (myelin-associated glycoprotein), *MBP* (myelin basic protein 1) and *MOBP* (myelin-associated oligodendrocyte basic protein) (Supplementary Fig. 1e, f).

### Signalling responsiveness and dependencies in *ACVR1* mutant DIPG cells

**Fig. 2:**
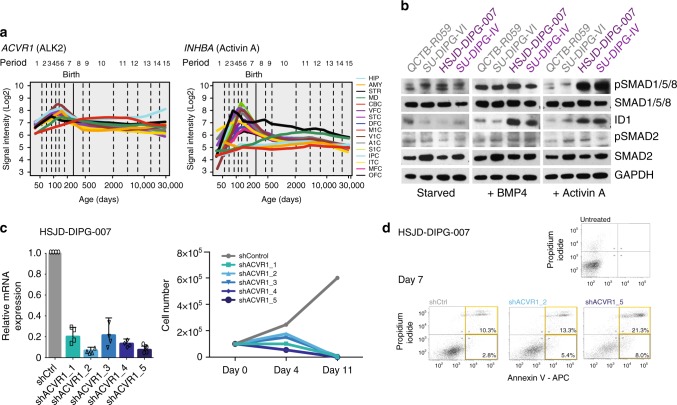
*ACVR1* in development and DIPG models. **a** Expression of *ACVR1* (ALK2) and *INHBA* (activin A) in anatomical structures of the developing and adult human brain (hbatlas.org). HIP, hippocampus; AMY, amygdala; STR, striatum; MD, mediodorsal nucleus of the thalamus; CBC, cerebellar cortex; VFC, ventrolateral prefrontal cortex; STC, posterior superior temporal cortex; DFC, dorsolateral prefrontal cortex; M1C, primary motor cortex; V1C, primary visual cortex; A1C, primary auditory cortex; S1C, primary somatosensory cortex; IPC, posterior inferior parietal cortex; ITC, inferior temporal cortex; MFC, medial prefrontal cortex; OFC, orbital prefrontal cortex. **b** Pathway activation by western blot of DIPG patient-derived cell cultures in response to growth factor starvation, and treatment with either BMP4 or Activin A. GAPDH is the loading control. **c** shRNA knockdown of *ACVR1* in HSJD-DIPG-007 cells. (left) qRT-PCR analysis of *ACVR1* expression in response to gene-specific constructs and shCTRL. (right) Effect of ACVR1 knockdown on cell number in vitro over 11 days. **d** Flow cytometry analysis by annexinV/propidium iodide staining of cell death in HSJD-DIPG-007 cells treated with shACVR1 constructs 2 and 5 and shCTRL. Early (bottom right) and late (top right) apoptotic cells are quantified

Mapping gene expression of *ACVR1* and *INHBA* (activin A) to publically available databases of neurodevelopment reveals a marked peak of activin A expression in the mid-foetal period at approximately 19 weeks post-conception, particularly in the neocortex and striatum, coinciding with that of a K27M gene expression signature thought to reflect the cellular origins of DIPG^26,27^. This is in contrast to *BMP4/6*, which are substantially reduced at the same time after peaking considerably earlier in development (Fig. 2a) (Supplementary Fig. 2a). Notably, a similar pattern of differential expression for *Acvr1*, *Inhba* and *Inhbb* was noted in the mouse pons/medulla compared to *Bmp4* and *Bmp6* (Supplementary Fig. 2b). Although employing an artificial exogenous source, using in vitro patient-derived DIPG models^10^, we observe a profound increase in phospho-SMAD1/5/8 in *ACVR1* mutant cells relative to wild-type only after activin A, rather than BMP4, stimulation in starved conditions, although the differential effects downstream on ID1 are similar with both ligands (Fig. 2b)(Supplementary Fig. 3). Knockdown of *ACVR1* by shRNA in H3.3 K27M, *ACVR1* R206H HSJD-DIPG-007 cells caused a dramatic reduction in cell viability after 4, and especially, 11 days, demonstrating the dependence of these cells on signalling through the receptor (Fig. 2c). Knockdown of *ACVR1* also resulted in significant cell death, with increased apoptosis seen by FACS (29.3% shACVR1_5 vs 13.1% shCTRL cells at 7 days) (Fig. 2d).

### Sensitivity of DIPG cells to ALK2 inhibitors

**Fig. 3:**
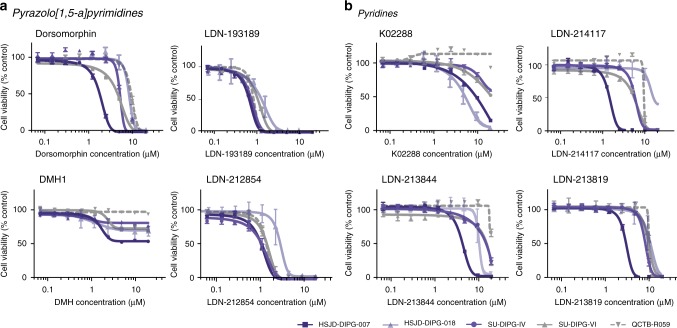
Screening of ALK2 inhibitors in vitro. Concentration-response curves for eight ALK2 inhibitors tested against three *ACVR1* mutant cell cultures (HSJD-DIPG-007 (R206H), SU-DIPG-IV (G328V), HSJD-DIPG-018 (R258G), purple) and two wild-type cultures (SU-DIPG-VI, QCTB-R059, grey). **a** Pyrazolo[1,5-a]pyrimidines—dorsomorphin, LDN-193189, DMH1, LDN-212854. **b** Pyridines—K02288, LDN-214117, LDN-213844, LDN-213819. Concentration of compound is plotted on a log scale (*x*-axis) against cell viability (*y*-axis). Mean plus standard deviation are plotted from at least *n* = 3 experiments

With *ACVR1* representing a potential drug target in DIPG, we next explored a range of pharmacological inhibitors of the protein product of the gene, ALK2, in a panel of *ACVR1* mutant and wild-type cells. We tested 11 compounds of distinct chemical series (Supplementary Fig. 4), including those based on the pyrazolo[1,5-a]pyrimidine scaffold (dorsomorphin, DMH1, LDN-193189, LDN-212854) (Fig. 3a), a series of pyridine compounds (K02288, LDN-214117, LDN-213844, LDN-213819) (Fig. 3b), as well as selected approved or clinically-investigated drugs with reported anti-ALK2 activity, including perhexiline maleate, momelotinib, and saracatinib (Supplementary Fig. 5). Whilst DIPG cells were largely insensitive to DMH1 and saracatinib, we observed GI_50_ values in the 1–10 µM range for the remaining compounds, with some selectivity against the R206H mutant HSJD-DIPG-007 cells, particularly for dorsomorphin (*p* = 0.0064) and the K02288 derivatives LDN-213844 (*p* = 0.0221) and LDN-213819 (*p* = 0.0004, all *t*-test) (Table 1). K02288 itself was most potent in the R258G mutant HSJD-DIPG-018 cells, which were conversely less sensitive than other mutants to the pyrazolo[1,5-a]pyrimidine compounds.

**Table 1:** Screening of ALK2 inhibitors

	HSJD-DIPG-007	SU-DIPG-IV	HSJD-DIPG-018	SU-DIPG-VI	QCTB-R059
*Histone H3*	*H3F3A* K27M	*HIST1H3B* K27M	*HIST1H3B* K27M	*H3F3A* K27M	*H3F3A* K27M
*ACVR1*	*ACVR1* R206H	*ACVR1* G328V	*ACVR1 R258G*	*ACVR1* wild-type	*ACVR1* wild-type
Dorsomorphin	1.85 ± 0.15 µM	6.21 ± 0.84 µM	8.70 ± 0.71 µM	5.01 ± 0.75 µM	10.36 ± 1.44 µM
DMH	>20 µM	>20 µM	>20 µM	>20 µM	>20 µM
LDN-193189	0.70 ± 0.09 µM	0.80 ± 0.02 µM	3.16 ± 0.23 µM	1.10 ± 0.20 µM	0.89 ± 0.01 µM
LDN-212854	1.25 ± 0.05 µM	1.30 ± 0.20 µM	3.22 ± 0.22 µM	1.70 ± 0.20 µM	1.70 ± 0.00 µM
K02288	9.10 ± 0.26 µM	>20 µM	14.34 ± 1.13 µM	>20 µM	>20 µM
LDN-214117	1.57 ± 0.03 µM	6.23 ± 0.30 µM	16.38 ± 0.79 µM	5.83 ± 0.18 µM	8.27 ± 0.33 µM
LDN-213844	3.67 ± 0.66 µM	12.79 ± 2.65 µM	15.44 ± 0.94 µM	12.70 ± 2.17 µM	>20 µM
LDN-213819	3.27 ± 0.15 µM	8.03 ± 0.31 µM	11.18 ± 0.61 µM	9.87 ± 0.94 µM	10.99 ± 1.63 µM
Perhexiline	4.07 ± 1.31 µM	4.70 ± 1.01 µM	6.11 ± 0.12 µM	4.90 ± 1.08 µM	5.33 ± 1.07 µM
Saracatinib	17.76 ± 0.56 µM	>20 µM	>20 µM	>20 µM	>20 µM
Momelotinib	7.32 ± 0.99 µM	14.23 ± 0.89 µM	9.50 ± 0.38 µM	10.42 ± 1.12 µM	15.94 ± 1.44 µM

Table of GI_50_ values for 11 compounds screened against four patient-derived DIPG cell cultures, with cell viability as the readout. Mean and standard error of the mean from at least *n* = 3 independent experiments are provided

**Fig. 4:**
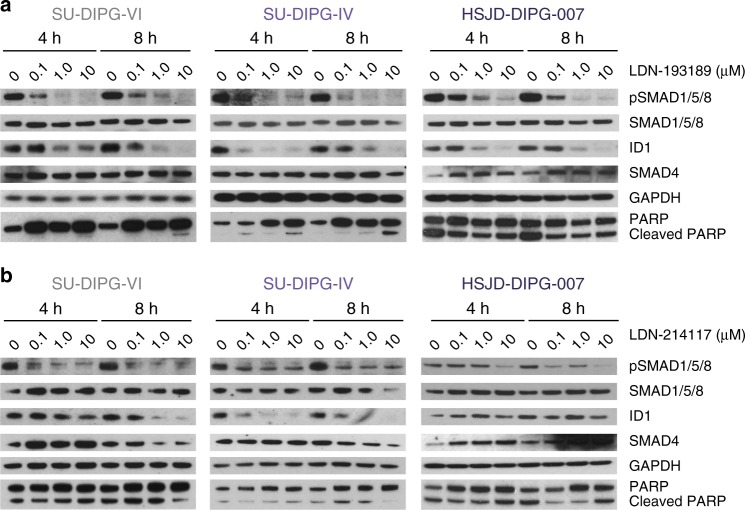
In vitro molecular pharmacology of LDN-193189 and LDN-214117. Western blot analysis of time- and concentration-dependent effects on downstream signalling in response to **a** LDN-193189 and **b** LDN-214117 in DIPG cells. Increasing concentrations (0–10 µM) at 4 and 8 h are shown for SU-DIPG-VI, HSJD-DIPG-IV and HSJD-DIPG-007. GAPDH is the loading control

The most potent compound was LDN-193189, with GI_50_ values in the range 0.70–3.16 µM, although little selectivity was observed between *ACVR1* mutant and wild-type cells. By contrast, the most selective compound was the pyridine LDN-214117, with GI_50_ values of 1.57 µM for R206H HSJD-DIPG-007 cells, 5.83–6.23 µM for G328V SU-DIPG-IV, and *ACVR1* wild-type (but high phospho-SMAD1/5/8 expressing) SU-DIPG-VI cells, and 8.27 µM for *ACVR1* wild-type QCTB-R059 cells (*p* < 0.0001, *t*-test) (Table 1). Both compounds were seen to inhibit signalling through phospho-SMAD1/5/8 and the downstream effector ID1, with the first effects seen at 4 h with 0.1 µM, but more profound consequences seen at 1.0 µM (Fig. 4a, b) (Supplementary Fig. 3). Induction of apoptosis evidenced by an increase in cleaved PARP was seen in *ACVR1* mutant, but not wild-type cells.

Such effects were not transient, with wash-out experiments for both compounds showing a persistent inhibition of downstream signalling 24 h post-treatment (Supplementary Fig. 6a, b). In the presence of ligands, there was no difference in GI50 values for LDN-193189 (Supplementary Fig. 6c), however, addition of Activin-A conferred enhanced sensitivity of R206H HSJD-DIPG-007 cells to LDN-214117 (Supplementary Fig. 6d). In order to assess whether treatment of DIPG with these compounds would afford a therapeutic window, we also assessed their effects on normal human astrocytes derived from the brainstem (NHA-BS). For both LDN-193189 (Supplementary Fig. 6e) and LDN-214177 (Supplementary Fig. 6f), there was a marked lack of sensitivity in the NHA-BS cells, with greater than 10-fold difference in effect on cell viability compared to the DIPG cultures.

### Pharmacokinetics and pharmacodynamics of ALK2 inhibitors

**Fig. 5:**
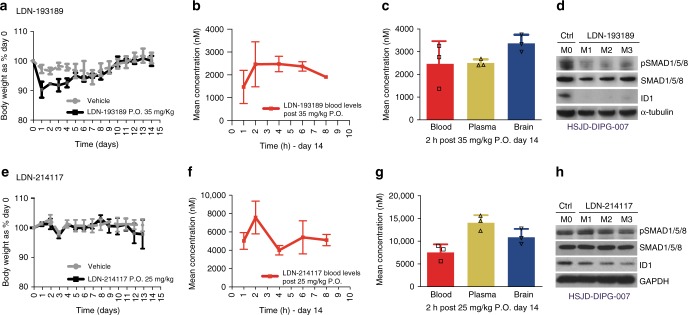
In vivo pharmacokinetics/pharmacodynamics of LDN-193189 (top) and LDN-214117 (bottom). **a**, **e** Tolerability in NOD.SCID mice exposed to daily oral treatment with inhibitor over 14 days, as assessed by body weight relative to day 0. Mean and standard deviation of 3 mice per group are plotted. **b**, **f** Blood levels of compounds up to 8 h post-exposure to a single oral dose of drug at day 14. Mean and standard deviation of 3 mice per group are plotted. **c**, **g** Barplot of compound concentrations in blood (red), plasma (yellow) and brain tissue (blue) 2 h post-exposure to a single oral dose of drug at day 14. Mean and standard deviation of 3 mice per group are plotted. **d**, **h** Western blot analysis of downstream pathway inhibition in three orthotopic HSJD-DIPG-007 tumours, after mice were treated with daily oral administration of compound, at the end of 28 days, compared with untreated controls. α-tubulin is the loading control

We next investigated which of the tested compounds possessed the desired biopharmaceutical profiles in terms of CNS penetration and in vivo effects on downstream signalling. Plasma drug exposure (area under the curve, AUC) was assessed for six compounds after oral and intravenous treatment, and bioavailability (F) was calculated. Those with the highest F were LDN-193189 (0.94), and to a lesser extent, LDN-214117 (0.75) (Supplementary Fig. 7). Both LDN-193189 and LDN-214117 were found to be well-tolerated in mice at 35 and 25 mg/kg, respectively, despite an initial weight loss with LDN-193189 (Fig. 5a, e). The concentrations of both compounds in the blood persisted over 8 h post oral administration (Fig. 5b, f), whilst importantly, brain levels were either even higher (LDN-193189 brain:plasma ratio = 1.34, 2 h post-dose, day 14) (Fig. 5c) or only slightly reduced compared with plasma (LDN-214117 brain:plasma ratio = 0.80, 2 h post-dose, day 14) (Fig. 5g). In both cases, the concentrations in mouse brain (3.37 and 10.94 µM) were considerably higher than the in vitro GI_50_ values observed (0.70 and 1.57 µM). Treatment at these concentrations also lead to a substantial reduction in pharmacodynamic biomarkers in HSJD-DIPG-007 xenograft tumours, showing a near-ablation of phospho-SMAD1/5/8 and ID1 with LDN-193189, and to a lesser extent with LDN-214117 (Fig. 5d, h; Supplementary Fig. 3).

### In vivo efficacy of targeting ALK2

**Fig. 6:**
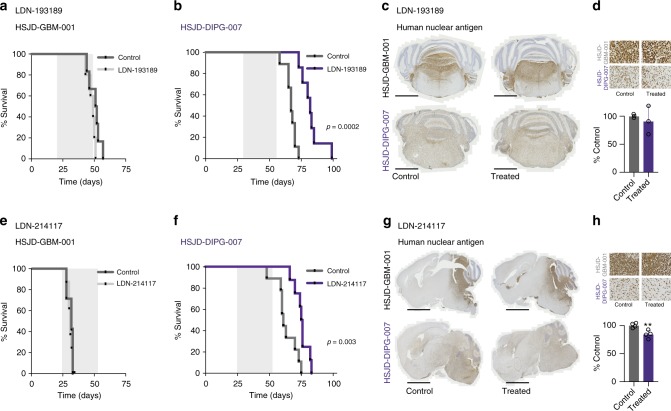
In vivo efficacy of LDN-193189 and LDN-214117 in orthotopic DIPG xenograft models. **a** Survival curves for mice (*n* = 7–10 per group) bearing HSJD-GBM-001 orthotopic xenografts, treated with LDN-193189, compared with vehicle-treated controls. **b** Survival curves for mice (*n* = 7–10 per group) bearing HSJD-DIPG-007 orthotopic xenografts, treated with LDN-193189, compared with vehicle-treated controls. **c** Immunohistochemistry of control and treated tumours for LDN-193189, in both models, for anti-human nuclear antigen (HNA). Scale bar = 1000 µM. **d** Magnified view of HNA staining (scale bar = 50 µM), and barplot quantifying cellularity by HNA-positive cells as a percentage of control. **e** Survival curves for mice (*n* = 7–10 per group) bearing HSJD-GBM-001 orthotopic xenografts, treated with LDN-214117, compared with vehicle-treated controls. **f** Survival curves for mice (*n* = 7–10 per group) bearing HSJD-DIPG-007 orthotopic xenografts, treated with LDN-214117, compared with vehicle-treated controls. **g** Immunohistochemistry of control and treated tumours for LDN-214117, in both models, for anti-human nuclear antigen (HNA). Scale bar = 1000 µM. **h** Magnified view of HNA staining (scale bar = 50 µM), and barplot quantifying cellularity by HNA-positive cells as a percentage of control. Mean and standard deviation plotted. **p < 0.01, *t*-test

Having identified two candidate compounds of distinct chemotypes with in vitro efficacy linked to effects on DIPG signalling, and brain penetration at doses sufficient to elicit a similar response in vivo, we next investigated their efficacy in orthotopic patient-derived xenografts. We treated established *ACVR1* mutant HSJD-DIPG-007 and H3/*ACVR1* wild-type (Supplementary Fig. 8a–c) brainstem xenografts at four and three weeks post-implantation, respectively, with either 25 mg/kg LDN-193189 or LDN-214177 for 28 days. No differences in survival were observed in wild-type control HSJD-GBM-001 tumours for either compound. By contrast, for both LDN-193189 and LDN-214177 we observed a significant prolongation of survival in *ACVR1* R206H-mutant HSJD-DIPG-007-bearing animals, amounting to a median benefit of 15 days in both cases (82 vs 67 days, *p* = 0.0002, LDN-193189; and 75 vs 61 days, *p* = 0.003, LDN-214117, log-rank test) (Fig. 6a, b, e, f). After 28 days, there was a decrease in cellularity of the treated *ACVR1* mutant tumours compared with controls, as assessed by counting of positive cells by immunohistochemical staining using anti-human nuclear antigen (*p* = 0.0079, LDN-214117, *t*-test) (Fig. 6c, d, g, h), though differences in proliferation (Ki67) (Supplementary Fig. 8d–g), and vascularisation (CD31) (Supplementary Fig. 8h–k) were not significant.

In summary, *ACVR1* mutations elicit a dependency of downstream signalling in DIPG cells than can be inhibited by single agents of multiple chemotypes of inhibitor compounds which penetrate orthotopic tumours at concentrations that produce cell death and survival benefit in orthotopic mouse models as single agents at well-tolerated dosages.

## Discussion

The discovery of somatic *ACVR1* mutations in DIPG identical to those found in the germline of FOP patients has not only highlighted unexpected links between neurodevelopment and chondrogenesis, but also allowed for the fast-tracked evaluation of specific ALK2 inhibitors developed for the control of heterotopic ossification in the context of childhood brain tumours^24^. Inhibition of ALK2, via pharmacologic or genetic means, led to inhibition of proliferation and induction of apoptosis, as well as a selective reduction in cell viability in patient-derived in vitro tumour models, and enhanced survival of mice bearing orthotopic *ACVR1*-mutant patient-derived DIPG xenografts in vivo.

A major challenge in the context of DIPG (unlike FOP) is the necessity for agents to penetrate the tumour behind what may be a particularly restrictive blood-brain barrier within the brainstem. Short-cutting drug development by using the reported off-target effects on ALK2 of drugs licensed for alternate targets and different indications holds promise given the urgency of the unmet clinical need, but appeared unwarranted for the compounds tested here (perhexiline maleate^28^, saracatinib^29^ and momelotinib^30^).

In our mouse models, derivatives of the earliest pyrazolo[1,5-a]pyrimidine- and pyridine-based compounds (LDN-193189^31^ and LDN-214117^32^, respectively) were found to be present within the brain at concentrations sufficient to ablate signalling via phospho-SMAD1/5/8 and ID1, biomarkers indicative of pathway inhibition and efficacy. With their good potency and selectivity, LDN-193189 and LDN-214117 are promising lead compounds. They have attractive physicochemical properties consistent with their CNS penetration and good pharmacokinetics due to low clearance and moderate volume of distribution. Notably, the distinct inhibitors were found to have differences in specificity for *ACVR1* mutant vs wild-type cultures, and determining such specificity profiles will be an important part of future drug development. Furthermore, the distinct ACVR1 mutations found in DIPG may themselves respond differently, with R206H more sensitive across a range of compounds, whilst the R258G mutant cells were largely insensitive to the pyrazolo[1,5-a]pyrimidines, though responded well to the pyridine-based compounds. There was also a clear therapeutic window demonstrated in respect of the lack of *sensitivity* in vitro of normal human astrocytes derived from the brainstem. Future development of inhibitors capable of producing effects across the whole spectrum of variants observed in DIPG patients will be critical to maximise patient benefit.

In FOP, *ACVR1* mutations confer aberrant responsiveness to the ligand activin A, an effect we demonstrate in DIPG, albeit using the addition of exogenous ligand in vitro. Of more clinical relevance is the observation that activin A expression also appears to peak at the predicted temperospatial origins of DIPG during neurodevelopment, suggestive of a direct mechanistic link to tumour development. Activin-A and *Acvr1* were also substantially elevated in the mouse pons/medulla compared to *Bmp4* and *Bmp6*, implying a feasible mechanism of receptor activation in our in vivo orthotopic xenograft experiments. Enhanced signalling by BMPs in the context of adult glioma is associated with a pro-differentiation programme^33^. *ACVR1* / H3.1 K27M mutant tumours appear to have a more astrocytic rather than oligodendroglial gene expression signature and morphological appearance^34^, although in terms of putative developmental origin it should be noted that activin A—ACVR1 signalling is associated with oligodendrocyte differentiation and myelination^16,17^, a developmental process actively occurring at the age and location at which DIPG arises. Notably, genes associated with oligodendrocyte differentiation were elevated in G328E/V/W mutant tumours compared to R206H/G356D. Although Activin A confers enhanced signalling via the same phospho-SMAD1/5/8-SMAD4 axis as BMPs, it remains possible that various non-canonical pathways may also be activated within this specific context. Gene expression signatures point towards a delicate balance associated with hypoxia^34^ and stem cell niche maintenance that remain unexplored.

Notably, not all *ACVR1* mutations appear to confer a better prognosis. The R206H mutation, which in the germline accounts for >95% of classical FOP patients^35^, does not have the striking predilection for H3.1 K27M mutations seen with the other variants, and being found in roughly equivalent numbers of H3.3 K27M tumours likely accounts for the extremely poor survival of these patients. That LDN-193189 and LDN-214117 were able to produce a survival benefit in vivo in a particularly aggressive H3.3 H27M/*ACVR1* R206H model of DIPG provides hope that such interventions may be more widely applicable to other genotypes.

Although statistically significant, the effect seen with these compounds was relatively modest, producing no cures in our mouse models, similar to other single agent intervention studies in DIPG^36–39^. Consequently, it is unclear how much benefit would be translated to the clinical setting of ALK2 inhibitors as single agents. Precisely how dependent DIPG cells are on the cell signalling conferred by *ACVR1* mutations at the time of presentation is not known, nor are the possible interactions of these inhibitors with the radiotherapy that all children with DIPG are currently given. Identification of co-segregation of *ACVR1* mutation and dysregulation in the PI3K pathway potentially points the way towards combinatorial approaches which may be tested in this subgroup of tumours. The Akt inhibitor perifosine was one of the earliest compounds evaluated in DIPG models^40^ and translated to the clinic^41^; sensitivity to the mTOR inhibitor TAK228 (MLN0128)^42^ and dual PI3K-mTOR inhibitor BKM120^36^ have also recently been reported and an ongoing stratified medicine trial in DIPG patients includes everolimus as one of three investigational arms (NCT02233049). The genetic dependence and preclinical efficacy demonstrated here with inhibitors of ALK2 raises the prospect of another class of compounds entering the clinical trials toolkit for children with this devastating disease.

## Methods

### Bioinformatic analysis

Curated gene-level copy number, expression and mutation data from DIPG patients were obtained from a recent meta-analysis of published and unpublished data^14^ and is available within the paediatric-specific implementation of the cBioPortal genomic data visualisation portal (pedcbioportal.org). Gene expression data from Agilent WG2.5, Affymetrix U133Plus2.0 or RNA sequencing were platform-centred, and log-transformed expression measures were combined and further normalised using pairwise loess normalisation. Gene Set Enrichment Analysis (software.broadinstitute.org/gsea) was performed using the GSEA java application based upon pairwise comparisons of the major subgroups in the merged dataset. Differential expression analysis was based on a Mann–Whitney *U* test of centred expression values between cases. Spatiotemporal gene expression data from developing and adult brain samples were obtained from the Human Brain Transcriptome project (hbatlas.org)^43^.

### Cell culture

All patient-derived material for cell culture was collected under IRB approval from Children’s Health Queensland Hospital. Patient-derived cultures HSJD-DIPG-007 (*H3F3A* K27M, *ACVR1* R206H), HSJD-DIPG-018 (*HIST1H3B* K27M, *ACVR1* R258G), SU-DIPG-IV (*HIST1H3B* K27M, *ACVR1* G328V), SU-DIPG-VI, QCTB-R059 (both *H3F3A* K27M, *ACVR1* wild-type), and HSJD-GBM-001 (H3*/ACVR1* wild-type) were grown in stem cell media consisting of Dulbecco’s Modified Eagles Medium: Nutrient Mixture F12 (DMEM/F12), Neurobasal-A Medium, HEPES Buffer Solution 1 M, sodium pyruvate solution 100 nM, non-essential amino acids solution 10 mM, Glutamax-I Supplement and Antibiotic-Antimycotic solution (all Thermo Fisher, Loughborough, UK). The media was supplemented with B-27 Supplement Minus Vitamin A, (Thermo Fisher), 20 ng/ml Human-EGF, 20 ng/ml Human-FGF-basic-154, 20 ng/ml Human-PDGF-AA, 20 ng/ml Human-PDGF-BB (all Shenandoah Biotech, Warwick, PA, USA) and 2 µg/ml Heparin Solution (0.2%, Stem Cell Technologies, Cambridge, UK). Normal Human astrocytes from the brainstem (NHA-BS) (Science Cell, #1840) were cultured according to manufacturer instructions. Briefly, cells were grown in complete astrocyte medium (Science Cell, #1840) using poly-lysine (Science Cell, #0413) coated flasks. Cell authenticity was verified using short tandem repeat (STR) DNA fingerprinting, and cells are available upon request.

### Western blot analysis

For growth factor starvation, cells were washed twice with PBS and then incubated for 1 h in stem cell medium without growth factors. Stem cell medium supplemented with BMP4 (10 ng/ml, Peprotech, London UK) or activin A (10 ng/ml, Thermo Fisher) was then added for 1 h and protein was collected. For treatment with LDN-193189 and LDN-214117, cells were incubated in complete media with vehicle or increasing concentrations of drug (0.1, 1, 10 µM) and protein was collected at 4 and 8 h post-treatment. For washout experiments, cells were incubated with 1 µM of LDN-193189 or LDN-214117 and either left in the culture media or washed out by media replacement for 2–24 h. Mouse brain samples were manually homogenised in protein cell lysis buffer. Samples were lysed by using lysis buffer (CST) containing phosphatase inhibitor cocktail (Sigma, Poole, UK) and protease inhibitor cocktail (Roche Diagnostics, Burgess Hill, UK). Following quantification using Pierce BCA Protein Assay Kit (Thermo Fisher), equal amounts of cell extracts were loaded for Western blot analysis. Membranes were incubated with primary antibody (1:1000) overnight at 4 °C, and horseradish peroxidase secondary antibody (Amersham Bioscience, Amersham, UK) for 1 h at room temperature. Signal was detected with ECL Prime western blotting detection agent (Amersham Biosciences), visualised using Hyperfilm ECL (Amersham Biosciences) and analysed using an X-ray film processor in accordance with standard protocols. Primary antibodies used were phospho-SMAD1/5/8 (CST#13820), phospho-SMAD2 (CST#3101), SMAD2 (CST#5339), PARP (CST#9542), α-tubulin (CST#2125), and GAPDH (CST#2118), all Cell Signalling (Danvers, MA, USA), and SMAD1/5/8 (SC#6031), SMAD4 (SC#7966), and ID1 (SC#488), all Santa Cruz Biotechnology (Dallas, TX, USA).

### *ACVR1* shRNA knockdown, qRT-PCR and apoptosis analysis

Lentiviral particles for shRNA knockdown of *ACVR1* were produced by co-transfecting the packaging vectors (pMD2.G, pMDLg/RRE and pRSV-Rev), and one individual shRNA expression vector into 293T cells. *ACVR1* shRNA vectors (Sigma) used were as follows: TRCN0000382239 (TRC2, shACVR1-1), TRCN0000315051 (TRC2, shACVR1-2), TRCN0000315051 (TRC2, shACVR1-3), TRCN0000000443 (TRC1, shACVR1-4) and TRCN0000000445 (TRC1, shACVR1-5) or the scrambled control shRNA vector (SHC002, shCTRL). Viral particles were concentrated using Lenti-x concentrator (Clontech, Mountain View, CA, USA) and resuspended in stem cell media. HSJD-DIPG-007 cells were plated onto laminin-coated plates and 24 h later transduced with either *ACVR1* or control shRNA lentiviral particles at a multiplicity of infection of 1 in the presence of polybrene (4 µg/ml). Viral medium was removed 24 h later and cells were selected with puromycin (5 µg/ml). On day 4 post-selection, cells were collected and RNA extracted for quantitation of *ACVR1* expression by qRT-PCR. RNA was reverse-transcribed using Superscript II Reverse Transcriptase (Thermo Fisher), followed by quantitative PCR using the Maxima SYBR Green/ROX qPCR (Thermo Fisher) on an Applied Biosystems 7900HT instrument. Reactions contained 0.5 μg RNA and individual measurements of human *ACVR1* and human *GAPDH* endogenous control were measured in triplicate. On days 4 and 11 cells were counted using a Vi-CELL XR (Beckman Coulter, High Wycombe, UK). Cellular apoptosis was measured using AnnexinV-APC Apoptosis Kit (BD Bioscience, Oxford, UK) on day 7 post-selection. Propidium Iodide (PI) was used as a vital dye. Cells were subjected to Annexin V/PI FACS analysis to assess the proportion of cells undergoing apoptosis. For FACS analyses, the stained cells were analyzed using BD machine (BD Biosciences).

### Compound efficacy assays

Cells were plated at a density of 2000–4000 cells/well on laminin-coated 96-well plates in a minimum of triplicates. After three days of incubation, compound was added to each well in concentrations from 0.07–20 µM and incubated at 37 °C, in 5% CO_2_, 95% humidity for eight days (192 h). For Activin A / BMP4 studies, cells were supplemented with media containing BMP4 (10 ng/ml, Peprotech, London UK) or Activin A (10 ng/ml, Thermo Fisher) 24 h prior to adding the drug. Drug was added in media supplemented with Activin A and BMP4 in concentrations from 0.07 to 20 µM and incubated at 37 °C, 5% CO_2_, 95% humidity for eight days (192 h). Dorsomorphin, DMH1, perhexiline maleate, LDN-193189, LDN-212854 and LDN-214117 were purchased from Sigma-Aldrich; saracatinib and momelotinib were purchased from Selleckchem (Houston, TX, USA); K02288 was purchased from BioFocus (Saffron Walden, UK); LDN-213844 (K03841b), LDN-212838 (K03449c) and K05907 were synthesised at the Structural Genomics Consortium, Oxford (see Supplementary Methods for chemical structures and synthesis). LDN-213819 was a kind gift from Paul Yu and Greg Cuny (Harvard University). Cell viability was assessed by the CellTiter-Glo luminescent cell viability assay (Promega, Madison, WI, USA) and GI_50_ values were calculated using GraphPad Prism version 6 as the concentration of compound required to reduce cell viability by 50%.

### Statement of compliance with ethical regulations guiding proper use of laboratory animals

All experiments utilizing mice were performed in accordance with institutional and European guidelines (EU Directive 2010/63/EU) and were approved by the local animal care and use committee (Comite Etico de Experimentacion Animal at Universidad de Barcelona, protocol 135/11).

### Pharmacokinetics

Nine-week-old female BALB/c mice were treated with LDN-193189, LDN-213844, LDN-212838, LDN-214117, K05907 and saracatinib at 5 mg/kg drug intravenously, 5 mg/kg drug by oral gavage, or vehicle. 20 µl blood was taken from the tail vein, spiked onto Whatman FTA DMPK-B cards and left to dry for at least 6 h. Female NOD.SCID mice were similarly treated with either 35 mg/kg LDN-193189, 25 mg/kg LDN-214117 or vehicle orally, with 20 µl tail vein blood taken at 1, 2, 4, 6 and 8 h after the first dose and prepared as before. Plasma and brain tissue samples were taken in triplicate at 2 h post-dose on day 14. Analysis was carried out by liquid chromatography-tandem mass spectrometry (LC-MS/MS) using a Waters Xevo TQS coupled with an Acquity UPLC H-class system (Waters, Herts, UK). Chromatography was carried out using a Phenomenex (Macclesfield, UK) C18 X-B column (2.6 µm, 50 mm × 2.1 mm). Data acquisition was performed using Targetlynx, version 4.1 and pharmacokinetic modelling was carried out using Phoenix WinNonlin version 6.3 (Certara, NJ, USA).

### In vivo efficacy studies

A single cell suspension of each culture was made the day before implantation and cultured overnight. On the implantation day, small tumourspheres in exponential growth were harvested by mild centrifugation. 3-week-old female NOD.SCID mice were anaesthetised with 100 mg/kg ketamine and 10 mg/kg xylazine and immobilized in a stereotaxic apparatus (Stoelting, Wood Dale, IL) at coordinates x+0.5 and y−5.4 from the bregma suture. 5 × 10^5^ HSJD-DIPG-007 or HSJD-GBM-001 tumourspheres (5 × 10^5^ cells), suspended in 5 μl matrigel (BD Biosciences) were injected at 3.1 mm depth (targeting the 4th ventricle) with a dull 22G needle attached to a 50 μl syringe (Hamilton, Bonaduz, Switzerland), using a stereotaxic arm. Mice were stratified (*n* = 7–10 per group) and treated with 25 mg/kg of either LDN-193189, LDN-214117 or vehicle for 28 days, starting 21–28 days post-inoculation. LDN-193189 was prepared each day in water whilst LDN-214117 was prepared in 10% DMSO diluted in saline. Mice were monitored by daily weighing and were sacrificed by cervical dislocation upon deterioration of condition or 20% weight loss from the maximum weight achieved, with tissue taken for further analysis. Mouse brains collected at the end of the efficacy study were processed for immunohistochemistry. Plasma and brain samples from treated and control mice were taken at 2 h post-dose at the end of the 28-day treatment for pharmacokinetic and pharmacodynamic analyses.

### Immunohistochemistry

PFA-fixed mouse brains were paraffin embedded and sectioned (4 µm) for immunohistochemical analysis and stained with haematoxylin and eosin (H&E). For immunohistochemistry, sodium citrate (pH 6.0) heat-mediated antigen retrieval was performed and staining was carried out using an antibody directed against human nuclear antigen (HNA) (Millipore, #4383, 1:100). Pressure antigen retrieval was performed and staining was carried out using antibodies directed against Ki67 (DAKO, #7240, 1:100), and CD31 (Abcam, #28364, 1:50). All primary antibodies were diluted into 1% Tris buffer solution with 0.05% Tween-20, except Ki67 which was diluted into Dako antibody diluent. Antibodies were incubated for 1 h at room temperature. Novocastra Novolink Polymer Detection Systems Kit (Leica Biosystem RE-7150) was used for the staining. Slides were then mounted using Leica CV Ultra mounting medium and slides were imaged using the high throughput-scanning microscope AxioScan Z1. Five fields of view were selected across sagittal and coronal cuts of the brain at 70% zoom and images quantified using Image J and application Cell Counter^44^.

### Statistics and reproducibility

Statistical analysis was carried out using R 3.3.0 (www.r-project.org) or GraphPad Prism version 6. Categorical comparisons of counts were carried out using Fisher's exact test, comparisons between groups of continuous variables employed Student’s t-test or ANOVA. Interventional in vitro experiments were carried out with at least *n* = 3 biological replicates. Differences in survival were analysed by the Kaplan–Meier method and significance determined by the log-rank test. All tests were two-sided and a *p*-value of less than 0.05 was considered significant. Multiple testing was accounted for using false discovery rate *q*-values or the Bonferroni adjustment.

### Reporting summary

Further information on research design is available in the Nature Research Reporting Summary linked to this article.

## Supplementary information

Supplementary Information

Supplementary Data 1

Description of Additional Supplementary Files

Reporting Summary

## Data Availability

The bioinformatic datasets generated analysed during the current study are available in the paediatric-specific implementation of the cBioPortal genomic data visualisation portal (http://www.pedcbioportal.org). Data showing gene expression differences in ACVR1 mutant vs wild-type 3 DIPG samples can be found on Supplementary Data 1. The authors declare that all other data supporting the findings of this study are available within the article, Supplementary Information files or available from the authors upon reasonable request.
