# Supplementary material for: ALK2 inhibitors display beneficial effects in preclinical models of ACVR1 mutant diffuse intrinsic pontine glioma
Source: Commun Biol. 2019 May 9;2:156. doi: 10.1038/s42003-019-0420-8 (PMC6509210; doi:10.1038/s42003-019-0420-8)
Supplement: Supplementary file 1 — Supplementary Information [file 42003_2019_420_MOESM1_ESM.pdf]

Supplementary Figures

Supplementary Figure 1

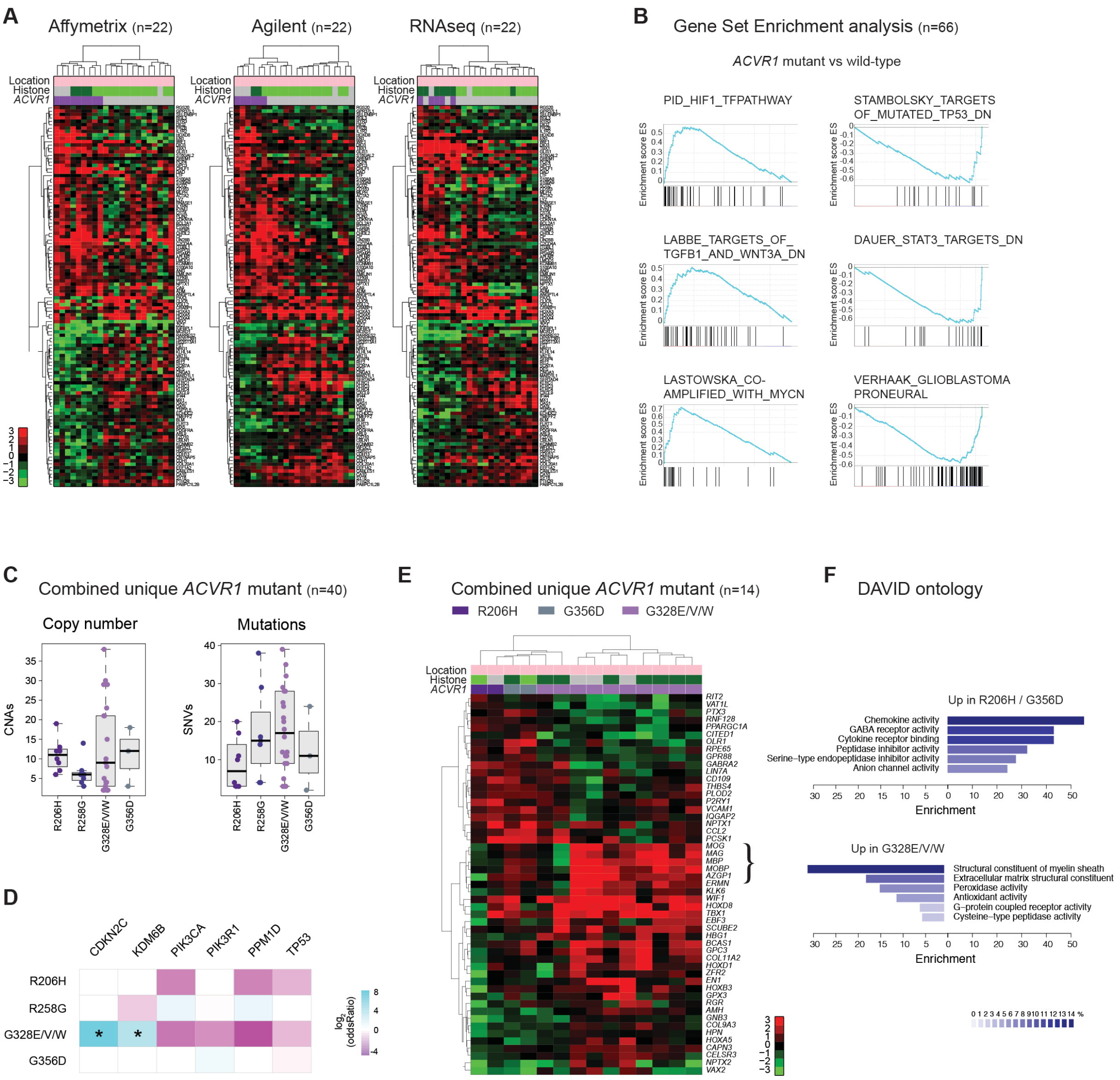

Supplementary Figure 1 – *Gene expression difference in ACVR1 mutant DIPG.* (A) Heatmap and hierarchical clustering of the top 100 of a consensus set of differentially expressed genes between *ACVR1* mutant and wild-type DIPGs, plotted independently from three platforms – Affymetrix, Agilent and RNAseq. (B) Gene set enrichment analysis from the combined gene expression dataset. Running enrichment scores are plotted of selected

enriched gene lists between *ACVR1* mutant and wild-type DIPGs. (C) Boxplots showing the number of DNA copy number alterations (left) and somatic mutations (right) in *ACVR1* mutant DIPGs, separated by specific variant. (D) Mutual exclusivity analysis. Pairwise Fishers exact tests are calculated for *CDKN2C, KDM6B, PIK3CA, PIK3R1, PPM1D* and *TP53*. Log_2-transformed odds ratios are plotted in a purple (negative correlation) to cyan (positive correlation) color scheme. White boxes represent no co-occuring events. *adjusted p<0.05. (E) Heatmap and hierarchical clustering of differentially expressed genes between distinct somatic variants of *ACVR1* in DIPG in the integrated expression dataset. (F) DAVID gene ontology analysis of differentially expressed genes between G328E/V/W variants and R206H/G356D. Bar length represents the enrichment score, coloured by adjusted p value.

Supplementary Figure 2

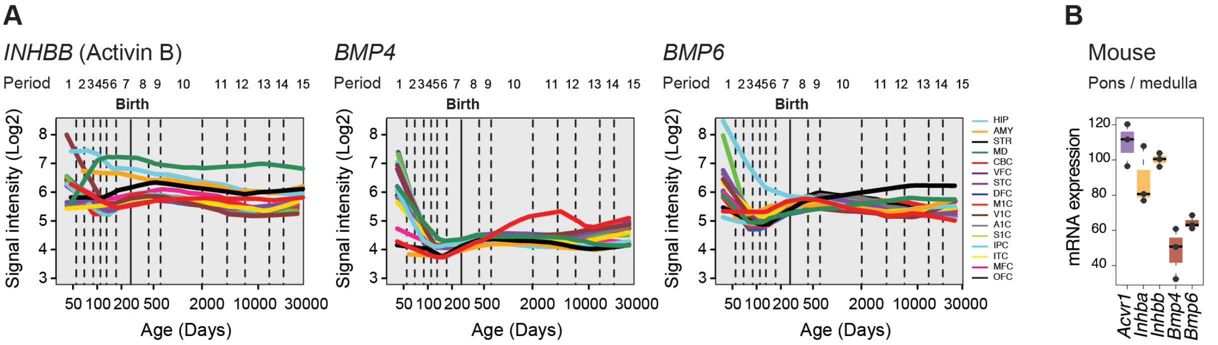

Supplementary Figure 2 – *Developmental gene expression of BMP/TGFβ receptor ligands.* (A) Expression of Activin B (*INHBB*), *BMP4* and *BMP6* in distinct anatomical structures in the developing and adult human brain, taken from the Human Brain Transcriptome project (hbatlas.org). HIP=hippocampus, AMY=amygdala, STR=striatum, MD=mediodorsal nucleus of the thalamus, CBC=cerebellar cortex, VFC=ventrolateral prefrontal cortex, STC=posterior superior temporal cortex, DFC=dorsolateral prefrontal cortex, M1C=primary motor cortex, V1C=primary visual cortex, A1C=primary auditory cortex, S1C=primary somatosensory cortex, IPC=posterior inferior parietal cortex, ITC=inferior temporal cortex, MFC=medial prefrontal cortex, OFC=orbital prefrontal cortex. (B) Boxplot of gene expression values for *Acvr1, Inhba, Inhbb, Bmp4* and *Bmp6* in the mouse pons/medulla, taken from GSE19709 (www.ncbi.nlm.nih.gov/geo).

Supplementary Figure 3

Fig2B (+Activin A)
Ab: pSMAD1/5/8 + pSMAD2

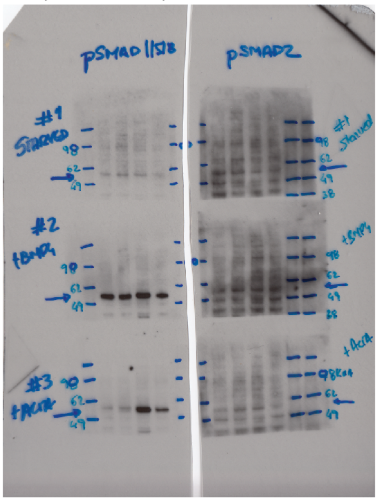

Fig2B (starved / +BMP4 / +Activin A)
Ab:ID1

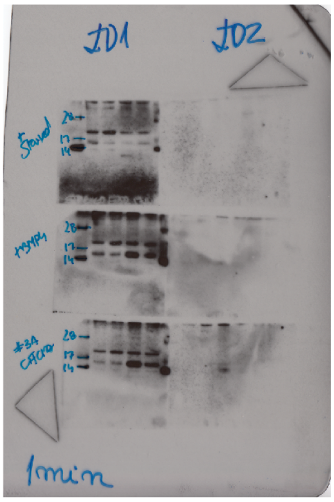

Fig2B (starved / +BMP4 / +Activin A)
Ab: SMAD1/5/8 + SMAD2

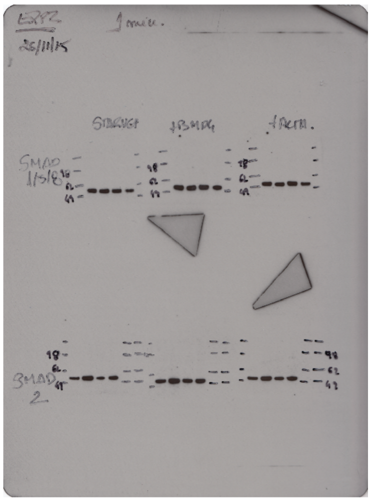

Fig2B (+Activin A)
Ab: GAPDH

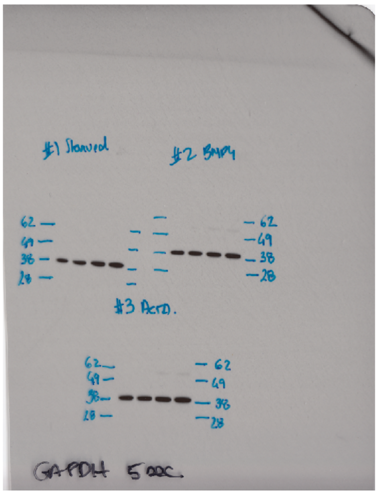

Fig2B (+BMP4 / +Activin A)
Ab: pSMAD1/5/8

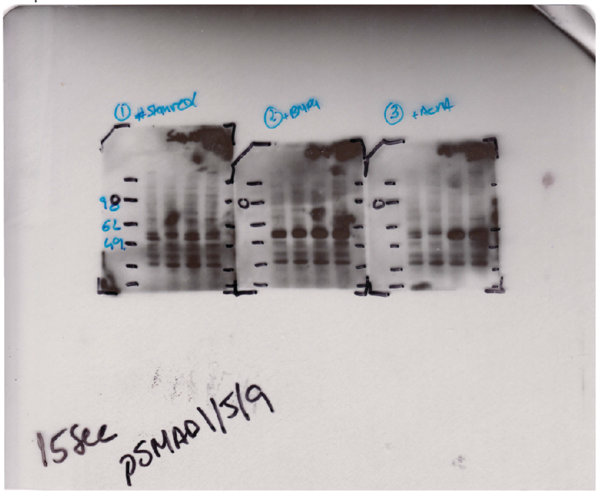

Supplementary Figure 3 – *Uncropped gel images for Western blot experiments.*

Supporting Figure 2B.

Supplementary Figure 3

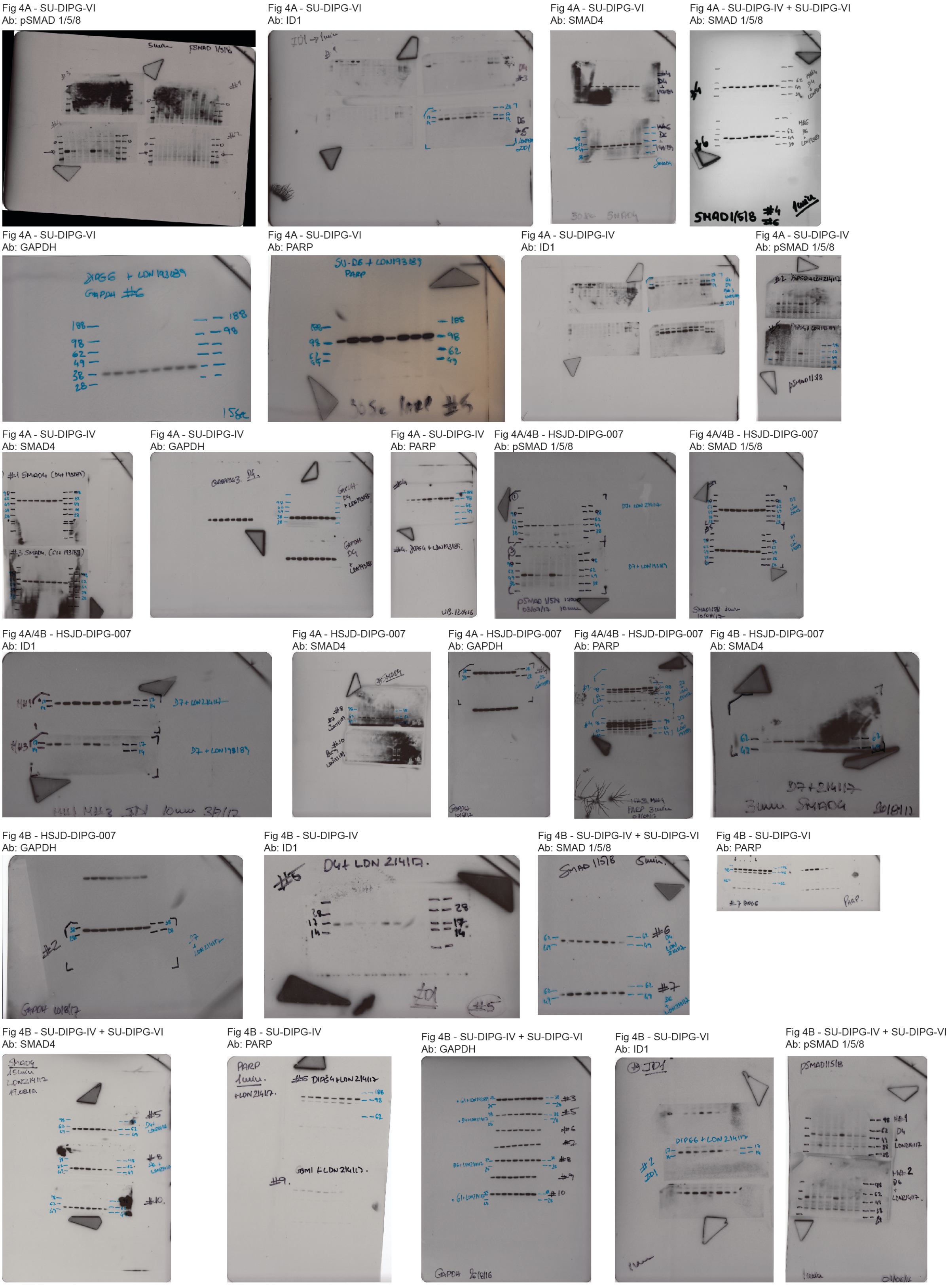

Supplementary Figure 3 – *Uncropped gel images for Western blot experiments.*

Supporting Figure 4,B.

Supplementary Figure 3

Fig5D
Ab: ID1

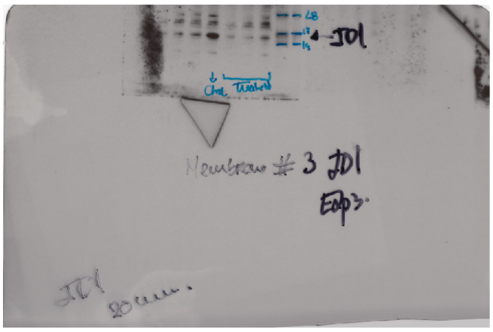

Fig5D
Ab: SMAD 1/5/8

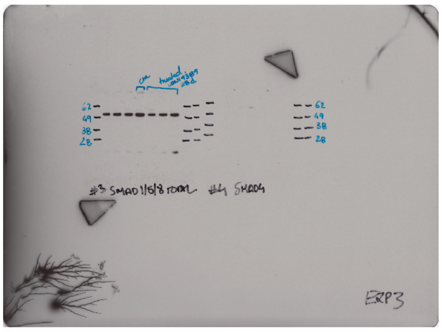

Fig5D
Ab: pSMAD1/5/8

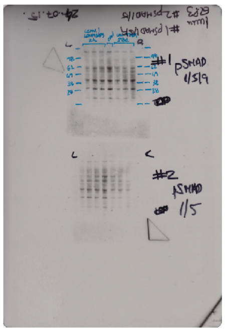

Fig5D
Ab: a-tubulin

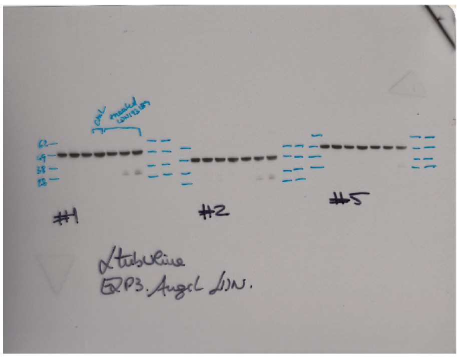

Fig5H
Ab: ID1

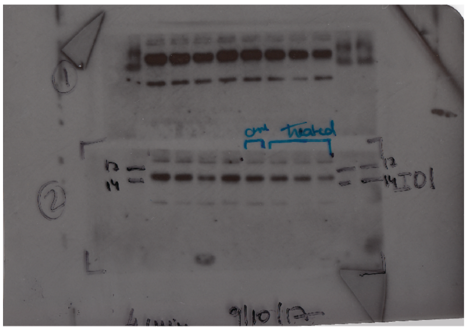

Fig5H
Ab: SMAD 1/5/8

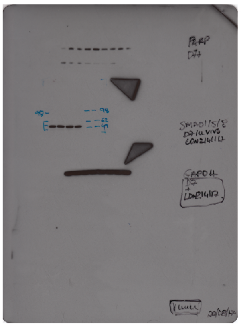

Fig5H
Ab: pSMAD 1/5/8

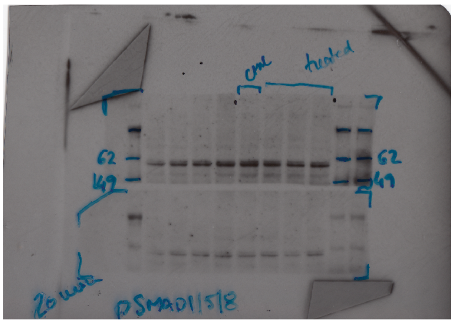

Fig5H
Ab: GAPDH

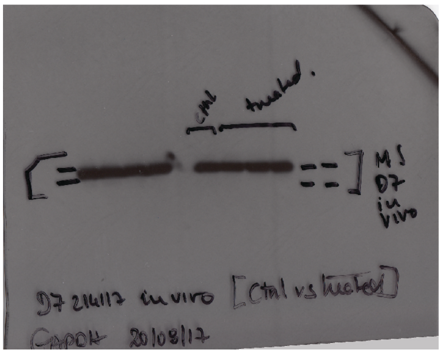

Supplementary Figure 3 – *Uncropped gel images for Western blot experiments.*

Supporting Figure 5D,H

Supplementary Figure 4

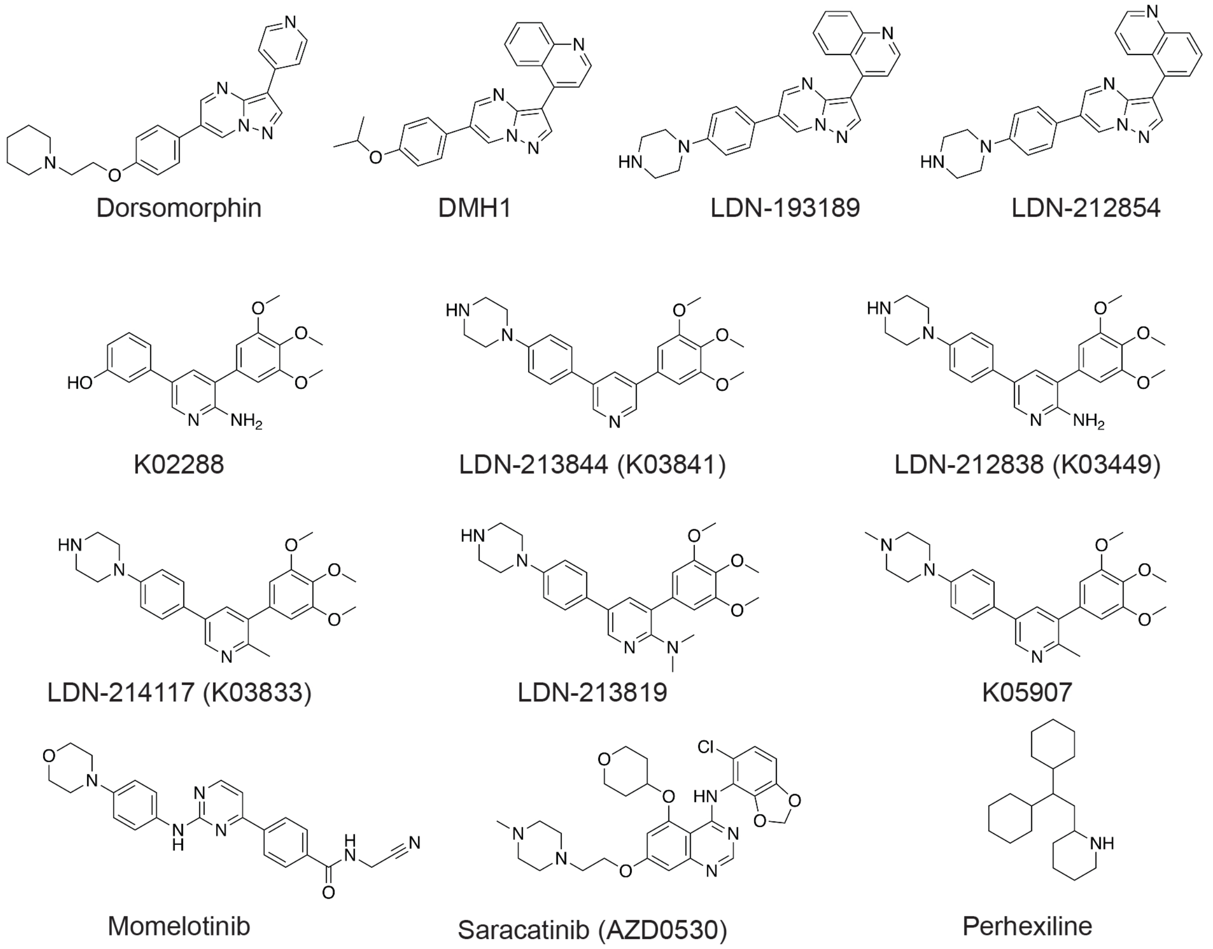

Supplementary Figure 4 – *Chemical structures of compounds tested.* Chemical structures given for dorsomorphin, DMH1, LDN-193189, LDN-212854, K02288, LDN-213844 (K03841), LDN-212838 (K03449), LDN-214117 (K03833), LDN-213819, K05907, momelotinib, saracatinib (AZD0530) and perhexiline.

Supplementary Figure 5

A

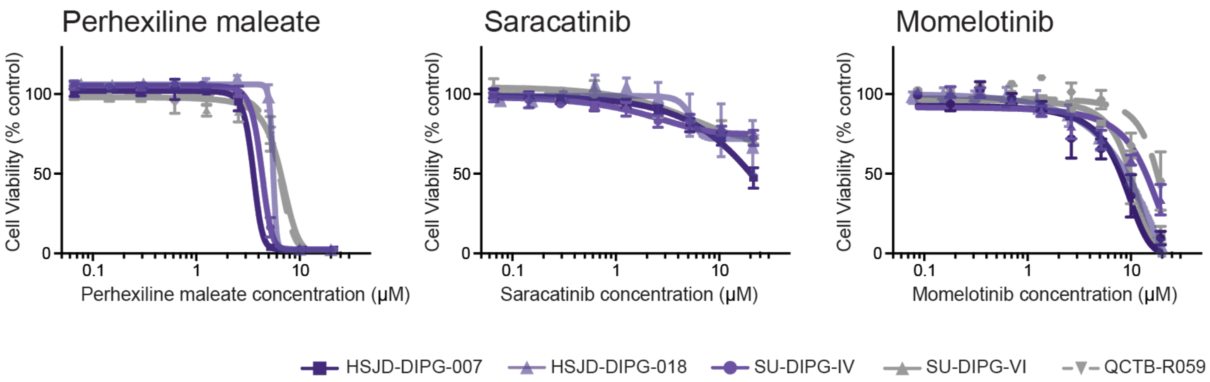

Supplementary Figure 5 – *Screening of ALK2 inhibitors in vitro*. Dose-response curves for perhexiline maleate, saracatinib and momelotinib tested against three *ACVR1* mutant cell cultures (HSJD-DIPG-007 (R206H), SU-DIPG-IV (G328V), HSJD-DIPG-018 (R258G), purple) and two wild-type cultures (SU-DIPG-VI, QCTB-R059, grey). Concentration of compound is plotted on a log scale (x axis) against cell viability (y axis). Mean plus standard error are plotted from at least n=3 experiments.

Supplementary Figure 6

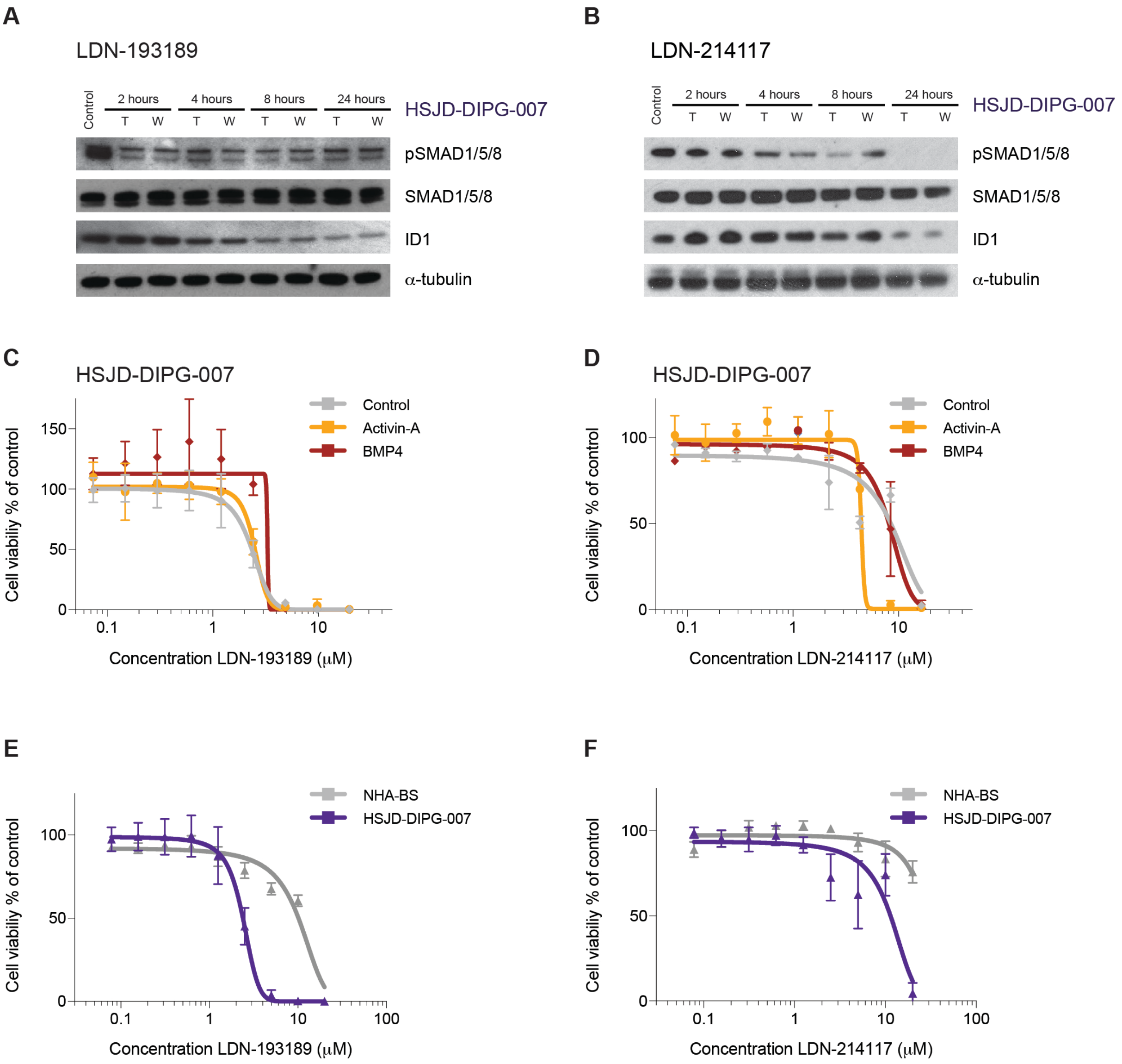

Supplementary Figure 6 – *In vitro molecular pharmacology of LDN-193189 and LDN-214117.* Western blot analysis of persistence of effects on downstream signalling in response to (A) LDN-193189 and (B) LDN-214117 in DIPG cells. HSJD-DIPG-007 cells were treated with 1.0 μM compound and either left in the culture media (T) or washed out by media replacement (W) for 2-24 hours. α-tubulin is the loading control. (C) Concentration-response curves for HSJD-DIPG-007 cells treated with LDN-193189 in the presence or absence of Activin-A and BMP4. Concentration of compound is plotted on a log scale (x axis) against cell viability (y axis). Mean plus standard deviation are plotted from at least n=3 experiments. (D)

Concentration-response curves for HSJD-DIPG-007 cells treated with LDN-214117 in the presence or absence of Activin-A and BMP4. Concentration of compound is plotted on a log scale (x axis) against cell viability (y axis). Mean plus standard deviation are plotted from at least n=3 experiments. (E) Concentration-response curves for HSJD-DIPG-007 and normal human astrocytes from the brainstem (NHA-BS) cells treated with LDN-193189. Concentration of compound is plotted on a log scale (x axis) against cell viability (y axis). Mean plus standard deviation are plotted from at least n=3 experiments. (F) Concentration-response curves for HSJD-DIPG-007 and normal human astrocytes from the brainstem (NHA-BS) cells treated with LDN-193189. Concentration of compound is plotted on a log scale (x axis) against cell viability (y axis). Mean plus standard deviation are plotted from at least n=3 experiments.

Supplementary Figure 7

A

Compounds	Route	Dose (mg/kg)	C_{max} (nmol/L)	AUC_{last} (h*nmol/L)	Cl (L/h)	$t_{1/2}$ (h)	Vz (L)	Vss (L)	F AUC last
LDN-193189	IV	5	1203	1412	0.139	2.58	0.465	0.60	-
	PO		354	1329	0.146	1.99	0.315	-	0.94
LDN-213844 (K03841)	IV	5	4146	6965	0.030	1.42	0.062	0.06	-
	PO		1278	3445	0.029	1.86	0.077	-	0.49
LDN-212838 (K03449)	IV	5	3684	6026	0.028	3.65	0.436	0.12	-
	PO		726	3117	0.021	5.03	0.450	-	0.52
LDN-214117 (K03833)	IV	5	4948	18430	0.013	3.73	0.068	0.06	-
	PO		1096	13773	0.013	3.91	0.077	-	0.75
K05907	IV	5	2845	6766	0.032	2.33	0.107	0.10	-
	PO		1008	6012	0.025	3.04	0.113	-	0.63
Saracatinib (AZD0530)	IV	5	3454	12612	0.013	7.23	0.135	0.09	-
	PO		1316	9187	0.014	6.92	0.140	-	0.73

B

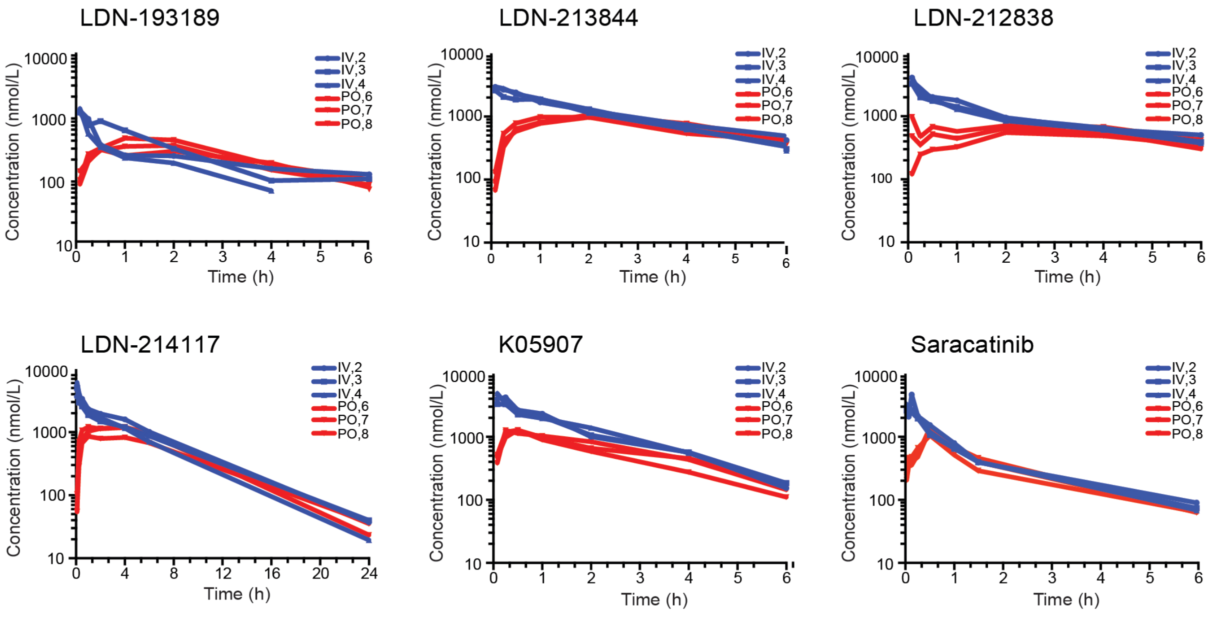

Supplementary Figure 7 – *In vivo pharmacokinetics of ALK2 inhibitors*. (A) 5mg/kg of six different compounds were administered orally or intravenously to three BALB/c mice, and a range of pharmacokinetic parameters measured, including peak serum concentration (C_{max}), area under the concentration:time curve up to last measurable concentration (AUC_{last}), clearance (Cl), half-life ($t_{1/2}$), apparent volume of distribution (V_z) and that at steady state (V_{ss}), and bioavailability (F). (B) Concentration (y axis, log scale):time (x axis) curves for all six compounds tested, both oral (red) and intravenous (blue) administration.

Supplementary Figure 8

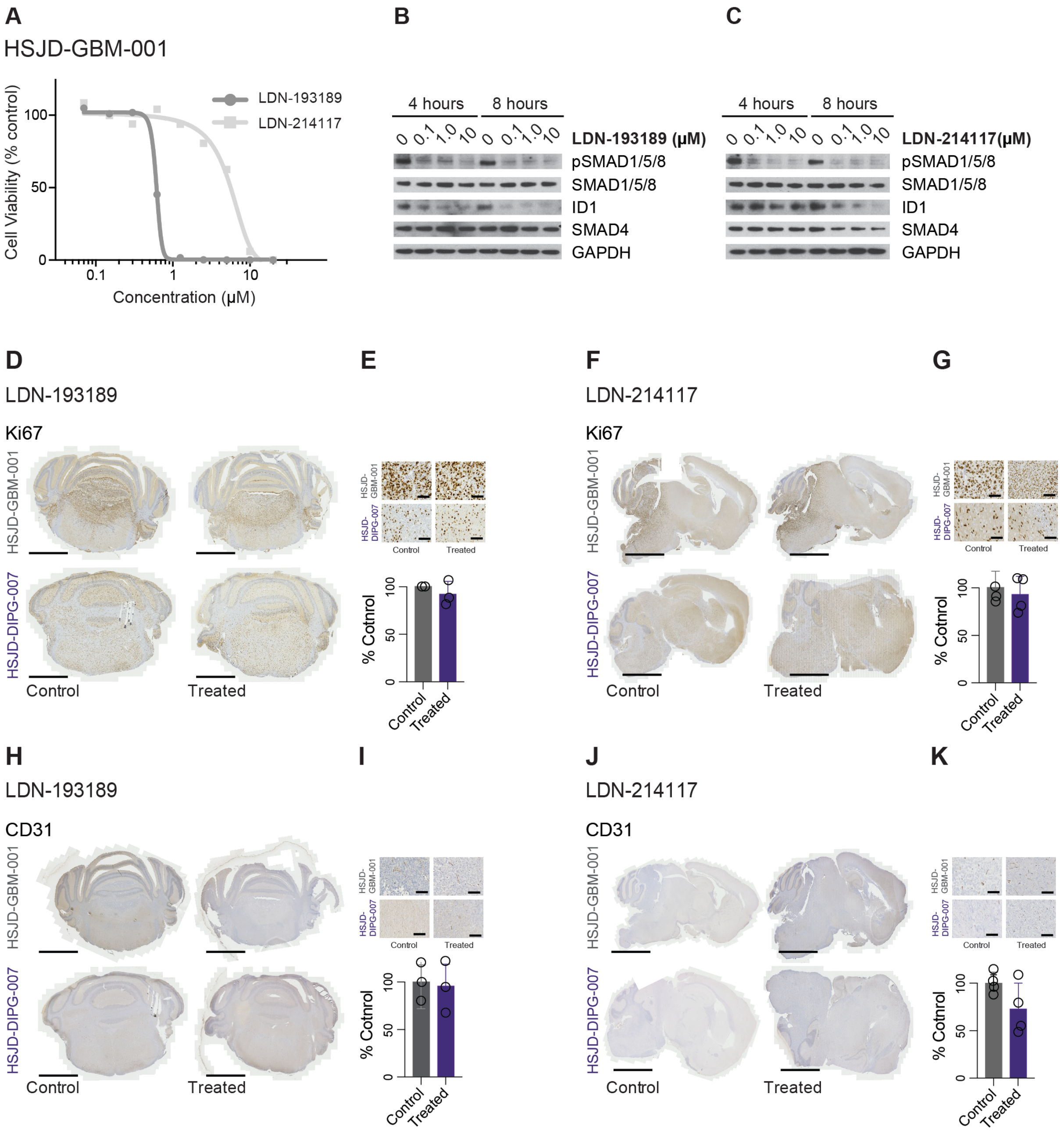

Supplementary Figure 8 – *In vitro and in vivo response and pharmacodynamics of LDN-193189 and LDN-214117.* (A) Dose-response curves for LDN-193189 and LDN-214117 in HSJD-GBM-001 cells. Concentration of compound is plotted on a log scale (x axis) against cell viability (y axis). Mean plus standard deviation are plotted from at least n=3 experiments. Western blot analysis of time- and concentration dependent effects on downstream signalling in response to (B) LDN-193189 and (C) LDN-214117 in HSJD-GBM-001 cells. Increasing concentrations (0-10 μM) at 4 and 8 hours are shown. GAPDH is the loading control. (D)

Immunohistochemistry of control and treated tumours for LDN-193189, in both HSJD-GBM-001 and HSJD-DIPG-007, for Ki67. Scale bar = 1000µM. (E) Magnified view of Ki67 staining (scale bar = 50µM), and barplot quantifying cellularity by Ki67-positive cells as a percentage of control. (F) Immunohistochemistry of control and treated tumours for LDN-214117, in both HSJD=GBM-001 and HSJD-DIPG-007, for Ki67. Scale bar = 1000µM. (G) Magnified view of Ki67 staining (scale bar = 50µM), and barplot quantifying cellularity by HNA-positive cells as a percentage of control. (H) Immunohistochemistry of control and treated tumours for LDN-193189, in both HSJD-GBM-001 and HSJD-DIPG-007, for CD31. Scale bar = 1000µM. (I) Magnified view of CD31 staining (scale bar = 50µM), and barplot quantifying cellularity by CD31-positive cells as a percentage of control. (J) Immunohistochemistry of control and treated tumours for LDN-214117, in both HSJD=GBM-001 and HSJD-DIPG-007, for CD31. Scale bar = 1000µM. (K) Magnified view of CD31 staining (scale bar = 50µM), and barplot quantifying cellularity by HNA-positive cells as a percentage of control.

Supplementary Methods

General Procedures

Starting materials, reagents and solvents (reagent or analytical grade, or dry over molecular sieves) were purchased from the following sources: Sigma Aldrich, VWR, Acros, Alfa Aesar, Fluorochem, Ark Pharm. All commercial chemicals and solvents were used without further purification or drying. All reactions were carried out under inert conditions, using Schlenk Technique, and if possible, monitored with thin layer chromatography on aluminium plates coated with 60 F_{254} silica gel. The plates were visualised using UV light and stained with aqueous $KMnO_4$. Flash column chromatography was performed on a Biotage Isolera One flash column chromatography platform. The structures of synthesized compounds were verified by ^{1}H-NMR, ^{13}C-NMR and mass spectrometry (LCMS). ^{1}H- and ^{13}C-NMR spectra were measured in $CDCl_3$ or DMSO-d_6 on a Bruker Avance spectrometer. Chemical shifts are reported in parts per million (ppm) using the residual peaks as internal standard, e.g. 7.26 (^{1}H) or 77.16 (^{13}C) ppm for $CDCl_3$. Mass spectra were obtained *via* LCMS analysis, carried out on a Waters system equipped with a Waters 2545 Binary Gradient Module, a Kinetex 5u EVO C18 100A 100 x 3.0 mm flash column, a Waters SQ Detector 2, Waters 2489 UV/Visible Detector and a Waters 2424 ELS Detector. The standard runtime was 3 min with a solvent gradient A/B = 95/5 to 5/95 (93 % H_2O, 5 % acetonitrile, and 2 % of 0.5 M ammonium acetate adjusted to pH 6 with glacial acetic acid. Solvent B: 18 % H_2O, 80 % acetonitrile, and 2 % of 0.5 M ammonium acetate adjusted to pH 6 with glacial acetic acid). Preparatory scale LCMS was performed on the same system equipped with a Kinetex 5u EVO C18 100A 150 x 21.2 mm flash column and a Waters 2767 Sample Manager.

Synthetic methods and analytical data

Precursors and Suzuki Coupling I

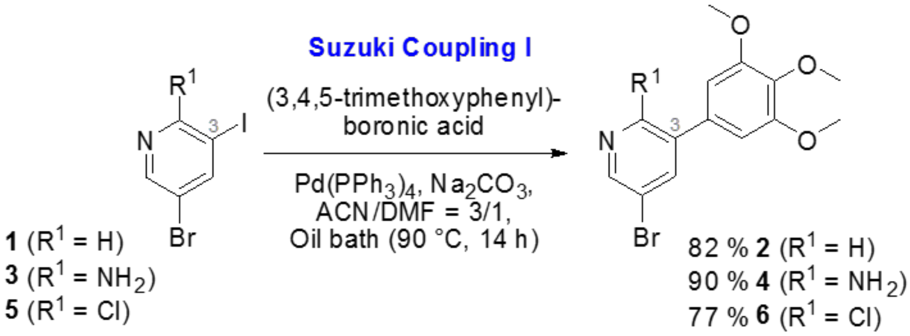

2-Amino-5-bromo-3-(3,4,5-trimethoxyphenyl)-pyridine

A mixture of 5-bromo-3-iodopyridin-2-amine (201 mg, 0.67 mmol, 1.0 equiv.), (3,4,5-trimethox-yphenyl)boronic acid (219 mg, 0.67 mmol, 1.0 equiv.), and $Pd(PPh_3)_4$ (93 mg, 0.08 mmol, 0.12 equiv.) was added to a sealed tube. The tube was evacuated and backfilled with argon (3 cycles). Acetonitrile (3.36 mL) and DMF (1.12 mL, v/v = 3/1) were added by syringe at room temperature, followed by (1 M) aqueous Na_2CO_3 (1.35 mL, 1.35 mmol, 2.0 equiv.). After being stirred at 90 °C for about 14 h, TLC still shows no more starting material. The reaction mixture was filtered through celite and concentrated *in vacuo.* The residue was purified by flash column chromatography (gradient: *c*Hexane to *c*Hexane/EtOAc = 4/6), yielding 5-bromo-3-(3,4,5-trimethoxyphenyl)pyridin-2-amine (210 mg, 0.62 mmol, 92 % yield) as a yellow solid.

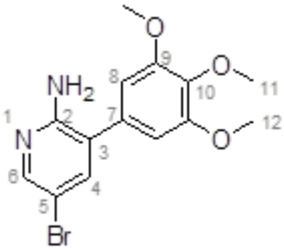

R_f (*c*Hexane/EtOAc = 6/4) = 0.29; **^{1}H-NMR** (400 MHz, $CDCl_3$) □ (ppm) = 8.09 (d, J = 2.3 Hz, 1H, H-6), 7.47 (d, J = 2.3 Hz, 1H, H-4), 6.61 (s, 2H, H-8), 4.66 (bs, 2H, NH_2), 3.89 (s, 3H,

H-11), 3.88 (s, 6H, H-12); **LCMS** (ES+): 340.03, 338.03 [M]+. The spectroscopic analysis was in agreement with the reported data.

5-Bromo-3-(3,4,5-trimethoxyphenyl)-pyridine

A mixture of 3-bromo-5-iodopyridine (785 mg, 2.77 mmol, 1.0 equiv.), (3,4,5-trimethoxy-phenyl)boronic acid (837 mg, 2.77 mmol, 1.0 equiv.), and $Pd(PPh_3)_4$ (383 mg, 0.33 mmol, 0.12 equiv.) were added to a sealed tube. The tube was evacuated and backfilled with argon (3 cycles). Acetonitrile (13.8 mL) and DMF (4.60 mL, v/v = 3/1) were added by syringe at room temperature, followed by (1 M) aqueous Na_2CO_3 (5.53 mL, 5.53 mmol, 2.0 equiv.). After being stirred at 90 °C for about 16 h, the reaction mixture was filtered through celite and concentrated *in vacuo.* The residue was purified by flash column chromatography (gradient: *c*Hexane to *c*Hexane/EtOAc = 4/6) and 3-bromo-5-(3,4,5-trimethoxyphenyl)pyridine (771 mg, 2.38 mmol, 86 % yield) was obtained as an off-white solid.

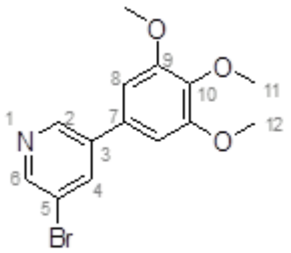

R_f (*c*Hexane/EtOAc = 6/4) = 0.59; **^{1}H-NMR** (400 MHz, $CDCl_3$) □ (ppm) = 8.72 (d, J = 1.9 Hz, 1H, H-2), 8.64 (d, J = 2.2 Hz, 1H, H-6), 7.97 (t, J = 2.1 Hz, 1H, H-4), 6.72 (s, 2H, H-8), 3.93 (s, 6H, H-12), 3.90 (s, 3H, H-11); **^{13}C-NMR** (400 MHz, $CDCl_3$) □ (ppm) = 154.0 (C-9), 149.5 (C-6), 146.5 (C-2), 138.9 (C-10), 138.6 (C-7), 136.9 (C-4), 132.2 (C-3), 121.0 (C-5), 104.7 (C-8), 61.1 (C-11), 56.5 (C-12); **LCMS** (ES+): 323.02 [M]+.

5-bromo-2-chloro-3-(3,4,5-trimethoxyphenyl)pyridine

A mixture of 5-bromo-2-chloro-3-iodopyridine (517 mg, 1.62 mmol, 1.0 equiv.), (3,4,5-tri-methoxyphenyl)boronic acid (430 mg, 1.62 mmol, 1.0 equiv.), and $Pd(PPh_3)_4$ (225 mg, 0.20 mmol, 0.12 equiv.) was added to a sealed tube. The tube was evacuated and backfilled with argon (3 cycles). Acetonitrile (8.12 mL) and DMF (2.71 mL, v/v = 3/1) were added by syringe at room temperature, followed by (1 M) aqueous Na_2CO_3 (3.25 mL, 3.25 mmol, 2.0 equiv.). After being stirred at 90 °C for about 14 h, the reaction mixture was filtered through celite and concentrated *in vacuo.* The residue was purified by flash column chromatography (gradtient: *c*Hexane/EtOAc = 8/2 to EtOAc pure), yielding 5-bromo-2-chloro-3-(3,4,5-trimethoxyphenyl)pyridine (450 mg, 1.26 mmol, 77 % yield) as a yellow solid.

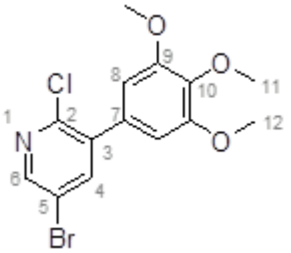

R_f (*c*Hexane/EtOAc = 7/3) = 0.46; **^{1}H-NMR** (400 MHz, $CDCl_3$) □ (ppm) = 8.45 (d, J = 2.4 Hz, 1H, H-6), 7.82 (d, J = 2.4 Hz, 1H, H-4), 6.63 (s, 2H, H-8), 3.91 (s, 3H, H-11), 3.90 (s, 6H, H-12); **^{13}C-NMR** (400 MHz, $CDCl_3$) □ (ppm) = 153.3 (C-9), 149.1 (C-6), 148.3 (C-2), 141.8 (C-4), 138.7 (C-10), 138.5 (C-7), 131.6 (C-3), 119.1 (C-5), 106.7 (C-8), 61.1 (C-11), 56.4 (C-12); **LCMS** (ES+): 356.98 [M]+.

Suzuki Coupling II to introduce free phenyl piperazine

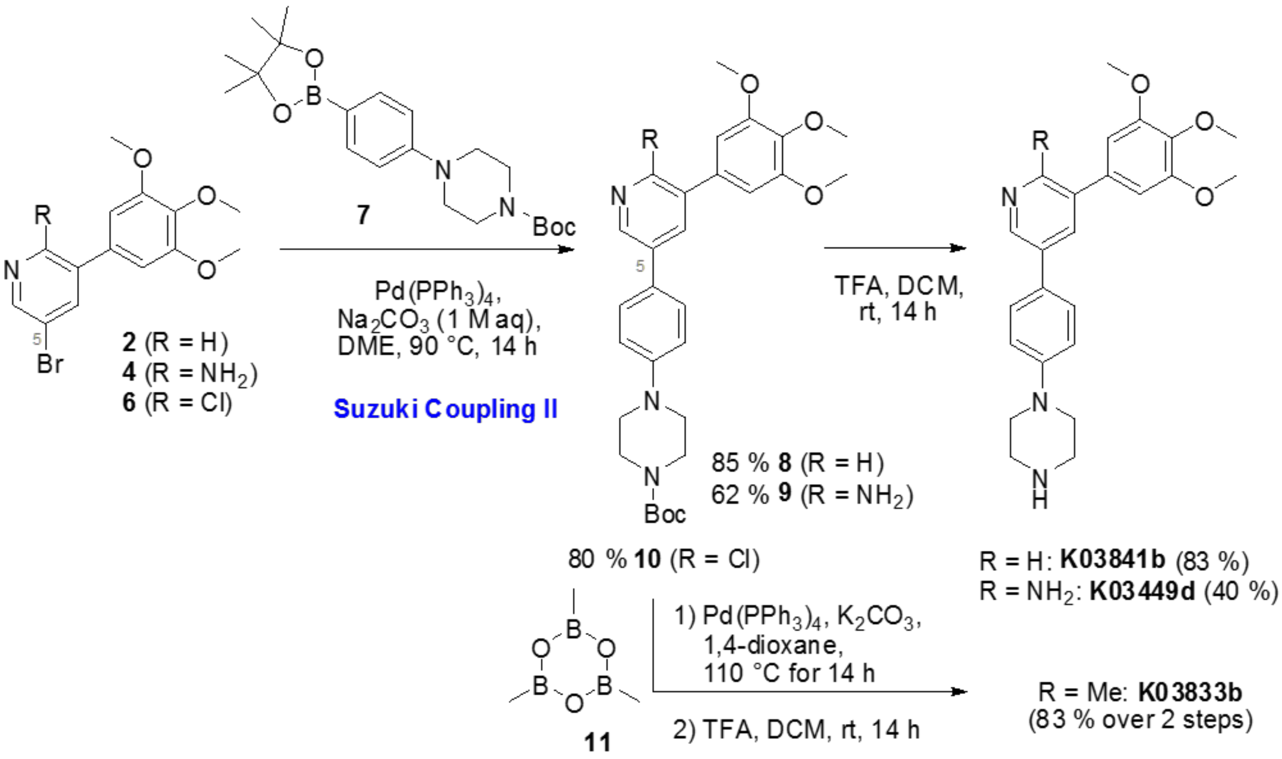

tert-butyl 4-(4-(5-(3,4,5-trimethoxyphenyl)pyridin-3-yl)phenyl)piperazine-1-carboxylate

To a solution of 3-bromo-5-(3,4,5-trimethoxyphenyl)pyridine (117 mg, 0.36 mmol, 1.0 equiv.), tert-butyl 4-(4-(4,4,5,5-tetramethyl-1,3,2-dioxaborolan-2-yl)phenyl)piperazine-1-carboxylate (154 mg, 0.40 mmol, 1.1 equiv.), and $Pd(PPh_3)_4$ (50 mg, 0.04 mmol, 0.12 equiv.) in DME (2.41 mL) was added (1 M) aqueous Na_2CO_3 (0.72 mL, 0.72 mmol, 2.0 equiv.). The reaction mixture was stirred under argon atmosphere at 90 °C for 14 h. The reaction mixture was filtered through celite and concentrated *in vacuo.* The residue was purified by flash column chromatography (gradient: *c*Hexane/EtOAc = 10/0 to 0/10), yielding tert-butyl 4-(4-(5-(3,4,5-trimethoxyphenyl)pyridin-3-yl)phenyl)piperazine-1-carboxylate (194 mg, 0.31 mmol, 85 % yield) as a yellow foam.

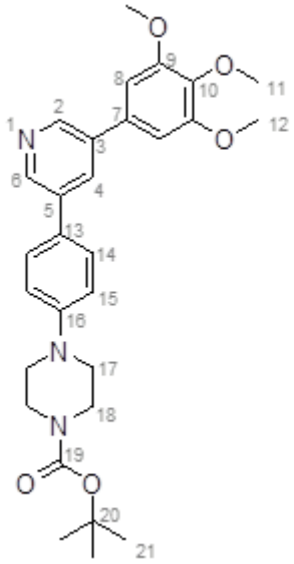

R_f (*c*Hexane/EtOAc = 6/4) = 0.15; **^{1}H-NMR** (400 MHz, $CDCl_3$) (ppm) = 8.78 (d, J = 2.2 Hz, 1H, H-6), 8.71 (d, J = 2.2 Hz, 1H, H-2), 7.94 (t, J = 2.2 Hz, 1H, H-4), 7.57 (d, J = 8.6 Hz, 2H, H-14), 7.04 (d, J = 8.6 Hz, 2H, H-15), 6.80 (s, 2H, H-8), 3.94 (s, 6H, H-12), 3.91 (s, 3H, H-11), 3.61 (m, 4H, H-17), 3.22 (m, 4H, H-18), 1.49 (s, 9H, H-21); **^{13}C-NMR** (400 MHz, $CDCl_3$) (ppm) = 154.9 (C-19), 153.9 (C-9), 151.4 (C-16), 146.7 (C-6), 146.3 (C-2), 138.5 (C-10), 136.9 (C-13), 136.3 (C-7), 134.0 (C-3), 129.0 (C-5), 128.7 (C-4), 128.1 (C-14), 116.8 (C-15), 104.7 (C-8), 80.2 (C-20), 61.1 (C-11), 56.4 (C-12), 49.0 (C-17), 43.7 (C-18), 28.6 (C-21).

tert-butyl 4-(4-(6-amino-5-(3,4,5-trimethoxyphenyl)pyridin-3-yl)phenyl)piperazine-1-carboxylate

To a solution of 5-bromo-3-(3,4,5-trimethoxyphenyl)pyridin-2-amine (232 mg, 0.68 mmol, 1.0 equiv.), tert-butyl 4-(4-(4,4,5,5-tetramethyl-1,3,2-dioxaborolan-2-yl)phenyl)piperazine-1-carboxylate (292 mg, 0.75 mmol, 1.1 equiv.), and $Pd(PPh_3)_4$ (95 mg, 0.08 mmol, 0.12 equiv.) in DME (4.56 mL) was added (1 M) aqueous Na_2CO_3 (1.37 mL, 1.37 mmol, 2.0 equiv.). The reaction mixture was stirred under argon atmosphere at 90 °C for 16 h. The reaction mixture was filtered through celite and concentrated *in vacuo.* The residue was purified by flash column chromatography (gradient: *c*Hexane/EtOAc = 8/2 to EtOAc pure), yielding tert-butyl 4-(4-(6-amino-5-(3,4,5-trimethoxyphenyl)pyridin-3-yl)phenyl)piperazine-1-carboxylate (220 mg, 0.42 mmol, 62 % yield) as a yellow foam.

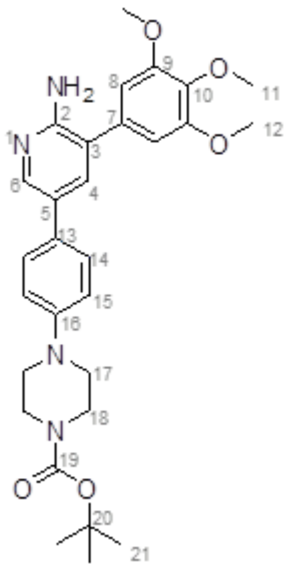

R$_f$ (*c*Hexane/EtOAc = 1/1) = 0.15; **^{1}H-NMR** (400 MHz, $CDCl_3$) □ (ppm) = 8.28 (d, J = 2.3 Hz, 1H, H-6), 7.57 (d, J = 2.3 Hz, 1H, H-4), 7.46 (d, J = 8.8 Hz, 2H, H-14), 6.99 (d, J = 8.8 Hz, 2H, H-15), 6.69 (s, 2H, H-8), 4.65 (bs, 2H, NH_2), 3.90 (s, 3H, H-11), 3.89 (s, 6H, H-12), 3.60 (m, 4H, H-17), 3.17 (m, 4H, H-18), 1.49 (s, 9H, H-21); **^{13}C-NMR** (400 MHz, $CDCl_3$) □ (ppm) = 154.7 (C-2), 154.6 (C-19), 153.7 (C-9), 153.7 (C-16), 150.4 (C-10), 144.9 (C-6), 135.9 (C-4), 133.6 (C-13), 133.1 (C-7), 130.0 (C-3), 127.0 (C-14), 121.8 (C-5), 116.9 (C-15), 105.8 (C-8), 79.9 (C-20), 61.0 (C-11), 56.2 (C-12), 49.3 (C-17), 43.8 (C-18), 28.5 (C-21).

tert-butyl 4-(4-(6-chloro-5-(3,4,5-trimethoxyphenyl)pyridin-3-yl)phenyl)piperazine-1-carboxylate

To a solution of 5-bromo-2-chloro-3-(3,4,5-trimethoxyphenyl)pyridine (198 mg, 0.44 mmol, 1.0 equiv.), tert-butyl 4-(4-(4,4,5,5-tetramethyl-1,3,2-dioxaborolan-2-yl)phenyl)piperazine-1-carboxylate (180 mg, 0.46 mmol, 1.1 equiv.), and $Pd(PPh_3)_4$ (61 mg, 0.05 mmol, 0.12 equiv.) in DME (2.95 mL) was added (1 M) aqueous Na_2CO_3 (0.88 mL, 0.88 mmol, 2.0 equiv.). The reaction mixture was stirred under argon atmosphere at 90 °C for 14 h. TLC shows no more SM. The reaction mixture was filtered and concentrated *in vacuo.* The residue was purified by flash column chromatography (gradient: *c*Hexane/EtOAc = 10/0 to 0/10), yielding tert-butyl 4-(4-(6-chloro-5-(3,4,5-trimethoxyphenyl)pyridin-3-yl)phenyl)piperazine-1-carboxylate (282 mg, 0.36 mmol, 80 % yield) as a yellow fluffy solid.

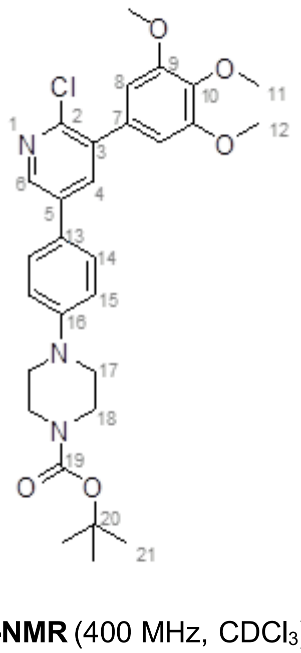

R_f (*c*Hexane/EtOAc = 7/3) = 0.41; **^{1}H-NMR** (400 MHz, $CDCl_3$) □ (ppm) = 8.56 (d, J = 2.5 Hz, 1H, H-6), 7.82 (d, J = 2.5 Hz, 1H, H-4), 7.52 (d, J = 8.8 Hz, 2H, H-14), 7.01 (d, J = 8.8 Hz, 2H, H-15), 6.69 (s, 2H, H-8), 3.92 (s, 3H, H-11), 3.90 (s, 6H, H-12), 3.60 (m, 4H, H-17), 3.21 (m, 4H, H-18), 1.49 (s, 9H, H-21); **^{13}C-NMR** (400 MHz, $CDCl_3$) □ (ppm) = 154.8 (C-19), 153.2 (C-9), 151.5 (C-16), 147.5 (C-2), 146.0 (C-6), 138.3 (C-10), 137.3 (C-4), 136.7 (C-7), 135.6

(C-13), 133.1 (C-5), 127.9 (C-14), 127.4 (C-3), 116.7 (C-15), 106.8 (C-8), 80.2 (C-20), 61.1 (C-11), 56.4 (C-12), 48.9 (C-17), 43.6 (C-18), 28.6 (C-21).

tert-butyl 4-(4-(6-methyl-5-(3,4,5-trimethoxyphenyl)pyridin-3-yl)phenyl)piperazine-1-carboxylate

A mixture of tert-butyl 4-(4-(6-chloro-5-(3,4,5-trimethoxyphenyl)pyridin-3-yl)phenyl)piperazine-1-carboxylate (99 mg, 0.18 mmol, 1.0 equiv.), 2,4,6-trimethyl-1,3,5,2,4,6-trioxatriborinane (0.10 mL, 0.73 mmol, 4.0 equiv.) and $Pd(PPh_3)_4$ (42 mg, 0.04 mmol, 0.2 equiv.) were added to a sealed tube. The tube was evacuated and backfilled with argon (3 cycles). Aqueous K_2CO_3 (1 M, 51 mg, 0.37 mmol, 2.0 equiv.) was added by syringe at room temperature. After being stirred at 110 °C for 12 h, the reaction mixture was filtered and concentrated *in vacuo.* The residue was purified by flash column chromatography (gradient: *c*Hexane/EtOAc = 9/1 to EtOAc pure), yielding tert-butyl 4-(4-(6-methyl-5-(3,4,5-trimethoxyphenyl)pyridin-3-yl)phenyl)piperazine-1-carboxylate (73 mg, 0.14 mmol, 77 % yield) as a colourless oil.

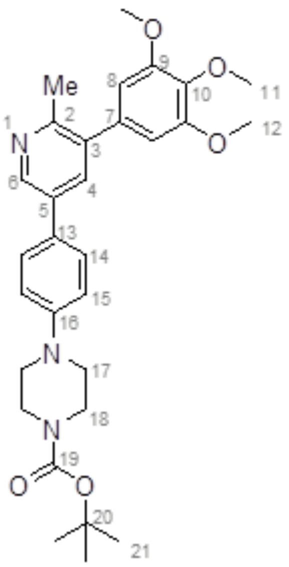

R$_f$ (*c*Hexane/EtOAc = 1/1) = 0.17; **^{1}H-NMR** (400 MHz, $CDCl_3$) □ (ppm) = 8.70 (d, J = 2.5 Hz, 1H, H-6), 7.69 (d, J = 2.3 Hz, 1H, H-4), 7.53 (d, J = 8.8 Hz, 2H, H-14), 7.01 (d, J = 8.8 Hz, 2H, H-15), 6.55 (s, 2H, H-8), 3.91 (s, 3H, H-11), 3.89 (s, 6H, H-12), 3.60 (m, 4H, H-17), 3.20 (m,

4H, H-18), 2.55 (m, 3H, Me), 1.49 (s, 9H, H-21). The spectroscopic analysis was in agreement with the reported data.

5-(4-(Piperazin-1-yl)phenyl)-3-(3,4,5-trimethoxyphenyl)-pyridin-2-amine

To a stirring solution of tert-butyl 4-(4-(6-amino-5-(3,4,5-trimethoxyphenyl)pyridin-3-yl)phenyl)piperazine-1-carboxylate (202 mg, 0.39 mmol, 1.0 equiv.) in dry DCM (78 mL, 0.005 M) at room temperature, was slowly added trifluoroacetic acid (TFA) (7.77 mL, 101 mmol, 260 equiv.) and the reaction mixture was stirred for 4 h. The mixture was concentrated under vacuum. The residue was suspended in EtOAc (10 mL), and then a saturated aqueous $NaHCO_3$ solution was added to adjust the pH to 7 at 0 °C. The mixture was extracted with EtOAc (3x). The combined organic phase was dried over anhydrous $MgSO_4$, filtered, and concentrated *in vacuo*. he remaining residue was subjected to flash column chromatography (gradient: DCM to DCM/MeOH = 9/1), yielding 5-(4-(piperazin-1-yl)phenyl)-3-(3,4,5-trimethoxyphenyl)pyridin-2-amine (66 mg, 0.16 mmol, 40 % yield) as an off-white foam.

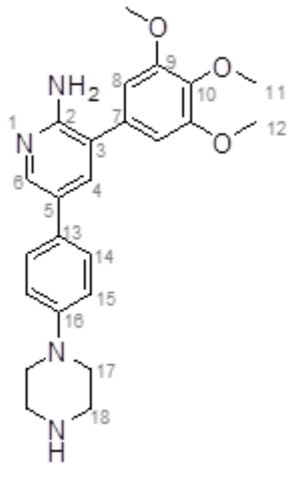

R_f (DCM/MeOH = 9/1) = 0.15; **^{1}H-NMR** (400 MHz, $CDCl_3$) □ (ppm) = 8.28 (d, J = 2.3 Hz, 1H, H-6), 7.58 (d, J = 2.3 Hz, 1H, H-4), 7.46 (d, J = 8.8 Hz, 2H, H-14), 6.99 (d, J = 8.8 Hz, 2H, H-15), 6.69 (s, 2H, H-8), 4.67 (bs, 2H, NH_2), 3.90 (s, 3H, H-11), 3.89 (s, 6H, H-12), 3.23 (m, 4H, H-17), 3.11 (m, 4H, H-18), 2.31 (bs, 1H, NH); **^{13}C-NMR** (400 MHz, $CDCl_3$) □ (ppm) = 154.6

(C-2), 153.9 (C-9), 150.9 (C-16), 144.8 (C-6), 137.8 (C-10), 136.1 (C-4), 133.6 (C-7), 129.6 (C-13), 127.8 (C-3), 127.1 (C-14), 122.0 (C-5), 116.6 (C-15), 105.9 (C-8), 61.1 (C-11), 56.4 (C-12), 50.1 (C-17), 45.9 (C-18); **LCMS** (ES+): 420.22 [M]+. The spectroscopic analysis was in agreement with the reported data.

1-(4-(5-(3,4,5-Trimethoxyphenyl)pyridin-3-yl)phenyl)-piperazine

To a stirring solution of the tert-butyl 4-(4-(5-(3,4,5-trimethoxyphenyl)pyridin-3-yl)phenyl)piperazine-1-carboxylate (101 mg, 0.20 mmol, 1.0 equiv.) in dry DCM (20 mL) at room temperature, was slowly added TFA (4.00 mL, 51.9 mmol, 260 equiv.) and the reaction mixture was stirred for 4 h. The mixture was concentrated under vacuum. The residue was suspended in EtOAc, and then a saturated aqueous $NaHCO_3$ solution was added to adjust the pH to 7 at 0 °C. The mixture was extracted with EtOAc. The combined organic phase was dried over anhydrous $MgSO_4$, filtered, and concentrated *in vacuo*. The remaining residue was subjected to flash column chromatography (gradient: DCM to DCM/MeOH = 9/1), yielding 1-(4-(5-(3,4,5-trimethoxyphenyl)pyridin-3-yl)phenyl)piperazine (78 mg, 0.17 mmol, 83 % yield) as a yellow foam.

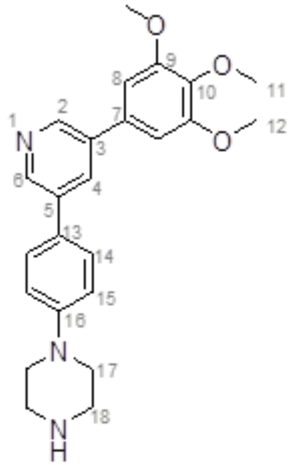

R_f (DCM/MeOH = 9/1) = 0.14; **^{1}H-NMR** (400 MHz, $CDCl_3$) □ (ppm) = 8.78 (d, J = 2.2 Hz, 1H, H-6), 8.71 (d, J = 2.2 Hz, 1H, H-2), 7.95 (d, J = 2.2 Hz, 1H, H-4), 7.57 (d, J = 8.7 Hz, 2H, H-14), 7.04 (d, J = 8.7 Hz, 2H, H-15), 6.80 (s, 2H, H-8), 3.94 (s, 6H, H-12), 3.91 (s, 3H, H-11),

3.27 (m, 4H, H-17), 3.11 (m, 4H, H-18), 1.99 (bs, 1H, NH); **^{13}C-NMR** (400 MHz, $CDCl_3$) □ (ppm) = 153.8 (C-9), 151.5 (C-16), 146.6 (C-6), 146.1 (C-2), 138.3 (C-10), 136.8 (C-3), 136.3 (C-5), 133.9 (C-7), 132.1 (C-4), 128.6 (C-13), 127.9 (C-14), 116.3 (C-15), 104.6 (C-8), 61.0 (C-11), 56.3 (C-12), 49.5 (C-17), 45.7 (C-18); **LCMS** (ES+): 405.21 [M]+. The spectroscopic analysis was in agreement with the reported data.

1-(4-(6-methyl-5-(3,4,5-trimethoxyphenyl)pyridin-3-yl)phenyl)piperazine

To a stirring solution of tert-butyl 4-(4-(6-methyl-5-(3,4,5-trimethoxyphenyl)pyridin-3-yl)phenyl)piperazine-1-carboxylate (55 mg, 0.11 mmol, 1.0 equiv.) in dry DCM (21 mL, 0.005 M) at room temperature, TFA (2.12 mL, 27.5 mmol, 260 equiv.) was slowly added and the reaction mixture was stirred for 4 h. The mixture was concentrated under vacuum. The residue was suspended in EtOAc, and then a saturated aqueous $NaHCO_3$ solution was added to adjust the pH to 7. The mixture was extracted with EtOAc (3x). The combined organic phase was dried over anhydrous $MgSO_4$, filtered, and concentrated *in vacuo*. The remaining residue was subjected to flash column chromatography (gradient: DCM to DCM/MeOH = 9/1), yielding 1-(4-(6-methyl-5-(3,4,5-trimethoxyphenyl)pyridin-3-yl)phenyl)piperazine (42 mg, 0.10 mmol, 93 % yield).

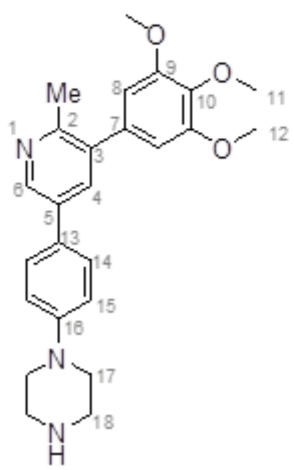

R_f (DCM/MeOH = 9/1) = 0.12; **^{1}H-NMR** (400 MHz, $CDCl_3$) □ (ppm) = 8.70 (d, J = 2.0 Hz, 1H, H-6), 7.69 (d, J = 2.0 Hz, 1H, H-4), 7.53 (d, J = 8.5 Hz, 2H, H-14), 7.01 (d, J = 8.5 Hz, 2H,

H-15), 6.56 (s, 2H, H-8), 3.92 (s, 3H, H-11), 3.88 (s, 6H, H-12), 3.26 (m, 4H, H-17), 3.11 (m, 4H, H-18), 3.01 (bs, 1H, NH), 2.55 (m, 4H, H-18); **LCMS** (ES+): 419.22 [M]+. The spectroscopic analysis was in agreement with the reported data.

Suzuki Coupling II to introduce methylated phenyl piperazine

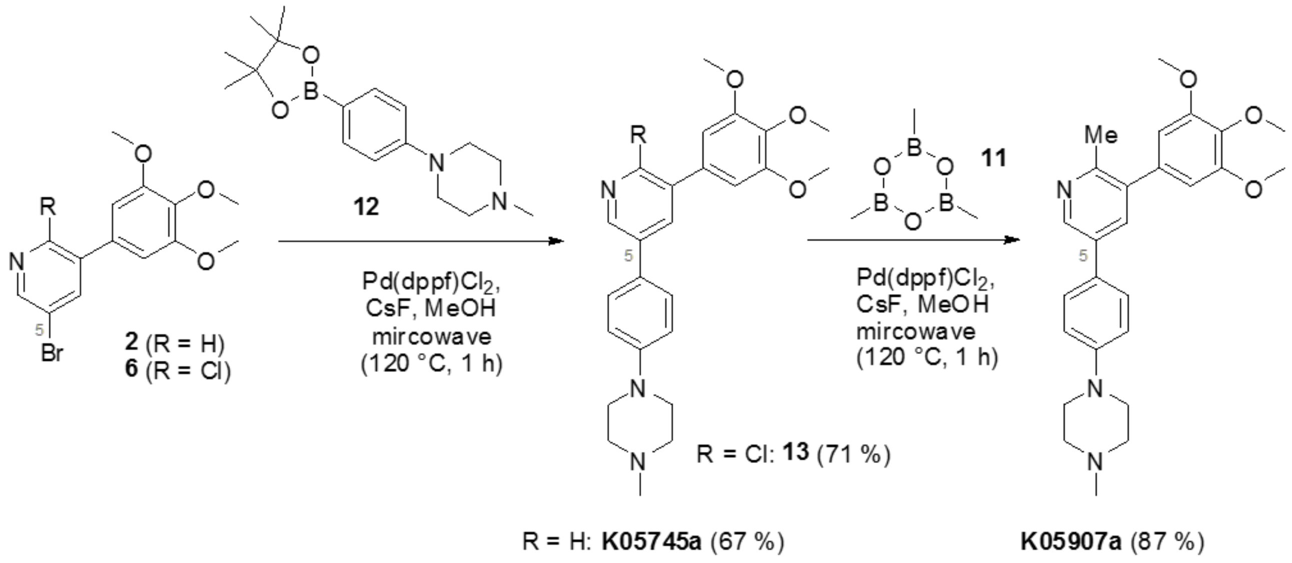

1-(4-(6-chloro-5-(3,4,5-trimethoxyphenyl)pyridin-3-yl)phenyl)-4-methylpiperazine

5-bromo-2-chloro-3-(3,4,5-trimethoxyphenyl)pyridine (149 mg, 0.33 mmol, 1.0 equiv.), 1-methyl-4-(4-(4,4,5,5-tetramethyl-1,3,2-dioxaborolan-2-yl)phenyl)piperazine (105 mg, 0.35 mmol, 1.1 equiv.) and CsF (177 mg, 1.16 mmol, 3.5 equiv.) were all added to a microwave vial, and MeOH (2.22 mL) was added. The mixture was thoroughly degassed, and $PdCl_2$(dppf) (12.2 mg, 0.02 mmol, 0.05 equiv.) was added. The reaction was heated in the microwave at 120 °C for 1 h, a black solution is formed. The reaction was partitioned between EtOAc and water. The organic phases were separated, dried over $MgSO_4$, filtered and evaporated to give a brown residue. The residue was purified by flash column chromatography (gradient: DCM to DCM/MeOH = 9/1), yielding 1-(4-(6-chloro-5-(3,4,5-

trimethoxyphenyl)pyridin-3-yl)phenyl)-4-methylpiperazine (108 mg, 0.24 mmol, 71 % yield) as a yellow sticky solid/oil.

R$_f$ (DCM/MeOH = 95/5) = 0.40; **^{1}H-NMR** (400 MHz, $CDCl_3$) □ (ppm) = 8.55 (d, J = 2.5 Hz, 1H, H-6), 7.81 (d, J = 2.5 Hz, 1H, H-4), 7.50 (d, J = 8.8 Hz, 2H, H-14), 7.00 (d, J = 8.8 Hz, 2H, H-15), 6.69 (s, 2H, H-8), 3.91 (s, 3H, H-11), 3.89 (s, 6H, H-12), 3.30 (m, 4H, H-17), 2.62 (m, 4H, H-18), 2.38 (s, 3H, H-19); **^{13}C-NMR** (400 MHz, $CDCl_3$) □ (ppm) = 153.2 (C-9), 151.4 (C-16), 147.3 (C-2), 145.9 (C-6), 138.2 (C-10), 137.2 (C-4), 136.6 (C-3), 135.7 (C-5), 133.1 (C-7), 127.9 (C-14), 126.8 (C-13), 116.2 (C-15), 106.8 (C-8), 61.1 (C-11), 56.4 (C-12), 54.9 (C-17), 48.4 (C-18), 46.1 (C-19); **LCMS** (ES+): 453.18 [M]+.

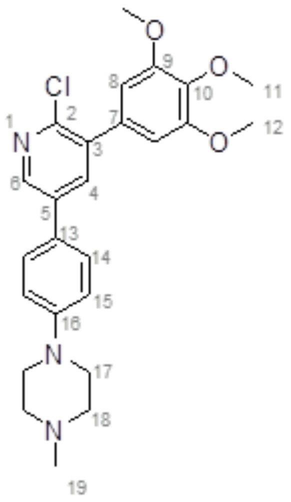

1-methyl-4-(4-(6-methyl-5-(3,4,5-trimethoxyphenyl)pyridin-3-yl)phenyl)piperazine

1-(4-(6-chloro-5-(3,4,5-trimethoxyphenyl)pyridin-3-yl)phenyl)-4-methylpiperazine (64 mg, 0.14 mmol, 1.0 equiv.), 2,4,6-trimethyl-1,3,5,2,4,6-trioxatriborinane (0.04 mL, 0.28 mmol, 4.0 equiv.), and CsF (75 mg, 0.49 mmol, 3.5 equiv.) were all added to a microwave vial, and MeOH (0.94 mL) was added. The mixture was thoroughly degassed, and $PdCl_2$(dppf) (5.16 mg, 7.05 □mol, 0.05 equiv.) was added. The reaction was heated in the microwave at 120 °C for 1 h, a black solution is formed. The reaction mixture was filtered and evaporated under reduced pressure. The residue was purified by flash column chromatography (gradient:

DCM to DCM/MeOH = 9/1), yielding 1-methyl-4-(4-(6-methyl-5-(3,4,5-trimethoxyphenyl)pyridin-3-yl)phenyl)piperazine (53 mg, 0.12 mmol, 87 % yield) as a brown-black sticky solid/oil. The remaining palladium residues were removed by subsequent filtration through a syringe filter and silica gel, thus obtaining a slightly yellow oil.

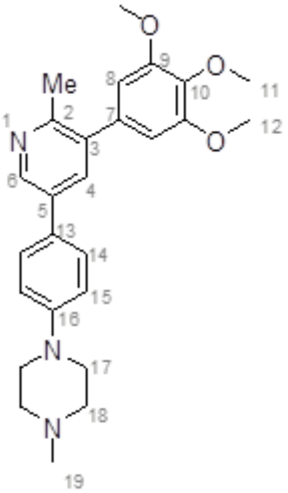

R_f (DCM/MeOH = 95/5) = 0.39; **^{1}H-NMR** (400 MHz, $CDCl_3$) □ (ppm) = 8.70 (d, J = 2.3 Hz, 1H, H-6), 7.69 (d, J = 2.3 Hz, 1H, H-4), 7.52 (d, J = 8.7 Hz, 2H, H-14), 7.01 (d, J = 8.7 Hz, 2H, H-15), 6.56 (s, 2H, H-8), 3.92 (s, 3H, H-11), 3.88 (s, 6H, H-12), 3.28 (m, 4H, H-17), 2.60 (m, 4H, H-18), 2.54 (s, 3H, Me), 2.37 (s, 3H, H-19); **^{13}C-NMR** (400 MHz, $CDCl_3$) □ (ppm) = 153.7 (C-9), 153.3 (C-16), 151.2 (C-2), 145.9 (C-6), 137.6 (C-10), 137.0 (C-3), 135.8 (C-5), 134.8 (C-4), 133.8 (C-7), 127.8 (C-13), 128.4 (C-14), 116.3 (C-15), 106.4 (C-8), 61.1 (C-11), 56.4 (C-12), 55.1 (C-17), 48.8 (C-18), 46.3 (C-19), 23.2 (Me); **LCMS** (ES+): 433.24 [M]+.

NMRs of Novel Final Compounds

1-(4-(6-chloro-5-(3,4,5-trimethoxyphenyl)pyridin-3-yl)phenyl)-4-methylpiperazine

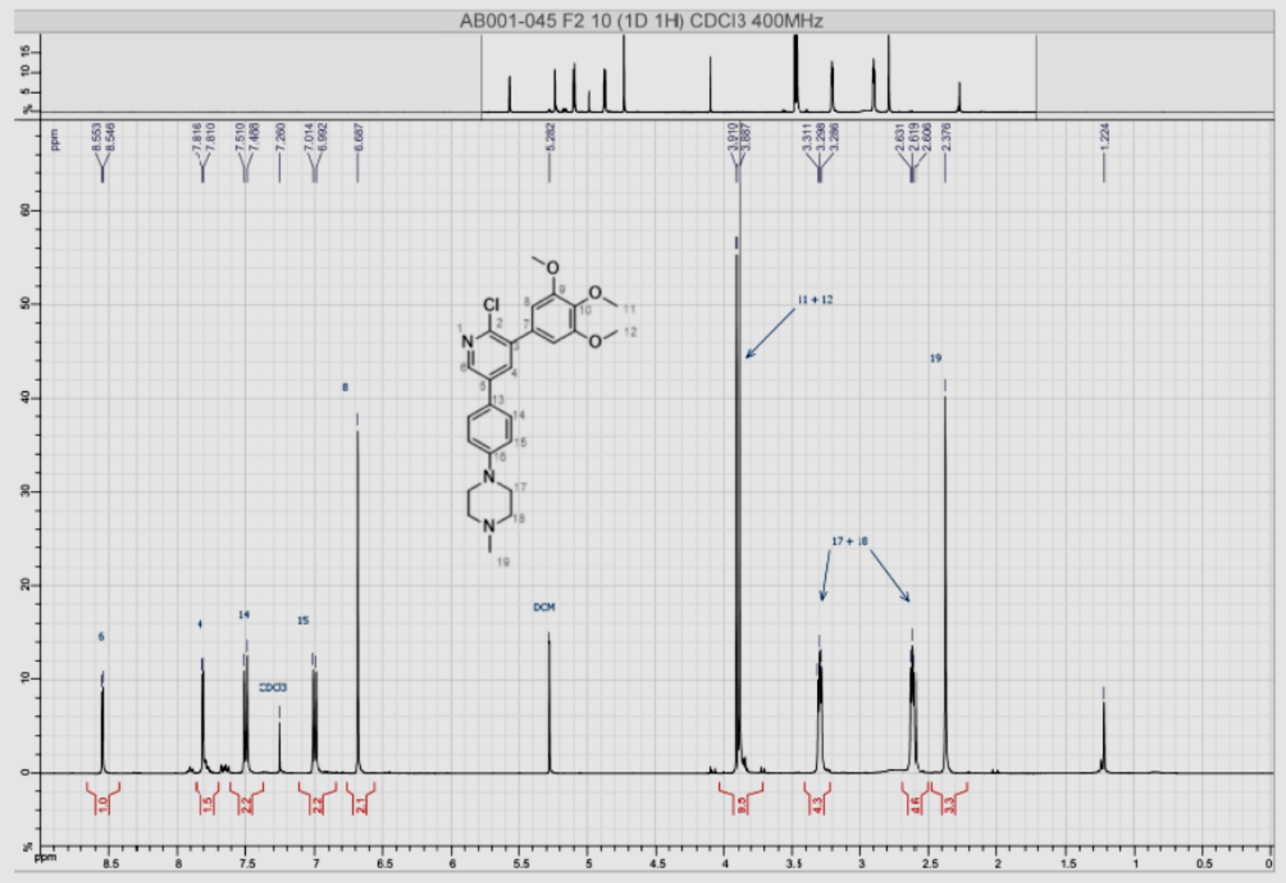

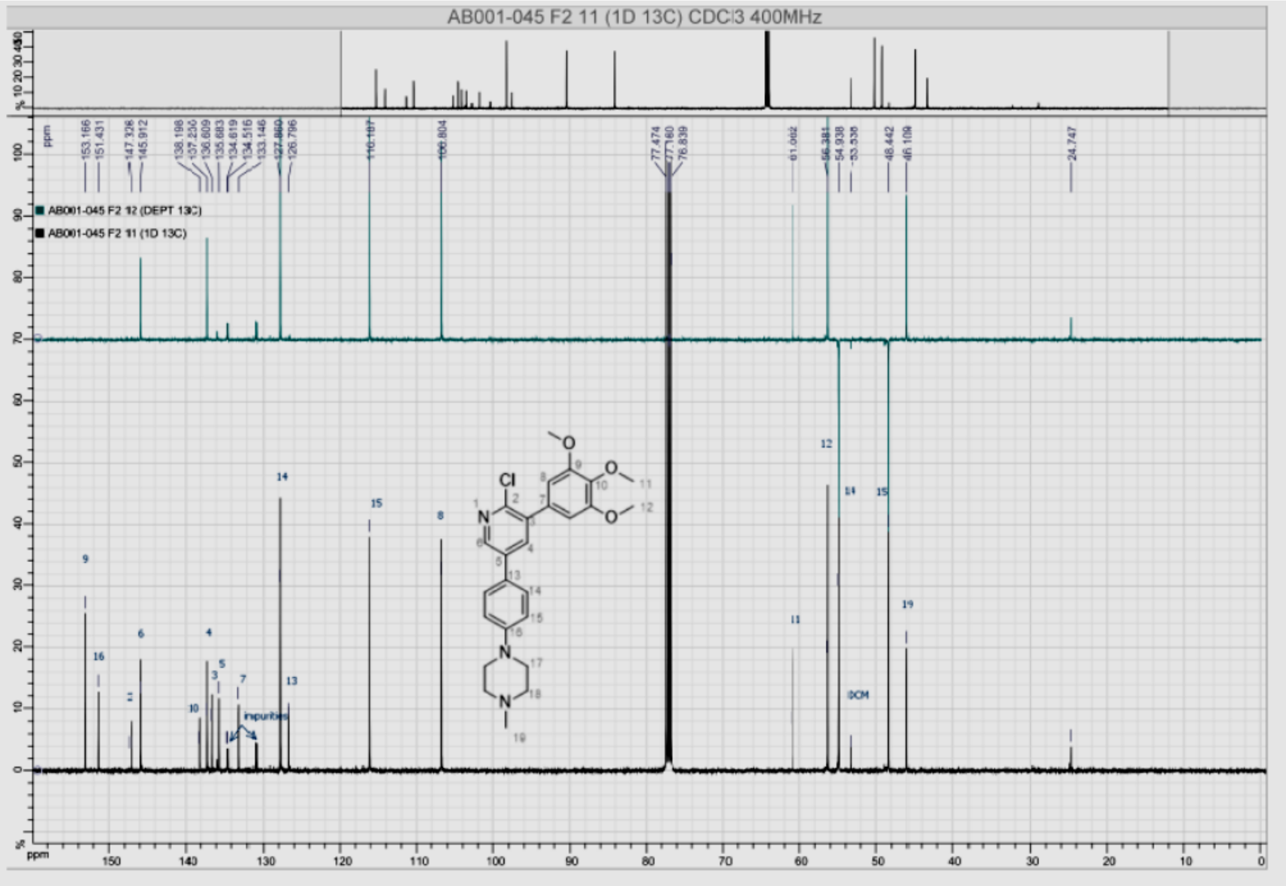
AB001-045 F2 11 (1D 13C) CDCl3 400MHz
AB001-045 F2 12 (DEPT 13C)
AB001-045 F2 11 (1D 13C)
impurities
DCM

1-methyl-4-(4-(6-methyl-5-(3,4,5-trimethoxyphenyl)pyridin-3-yl)phenyl)piperazine

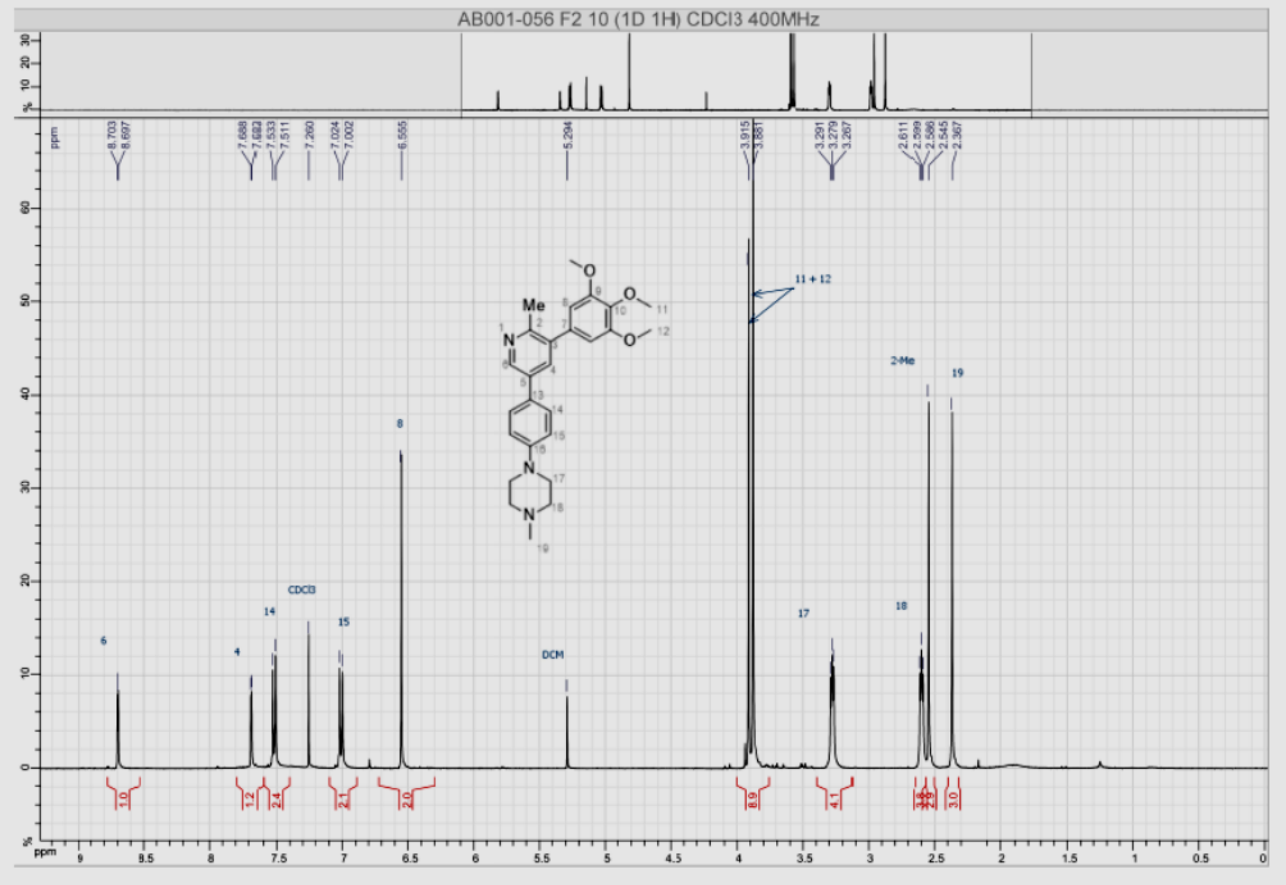

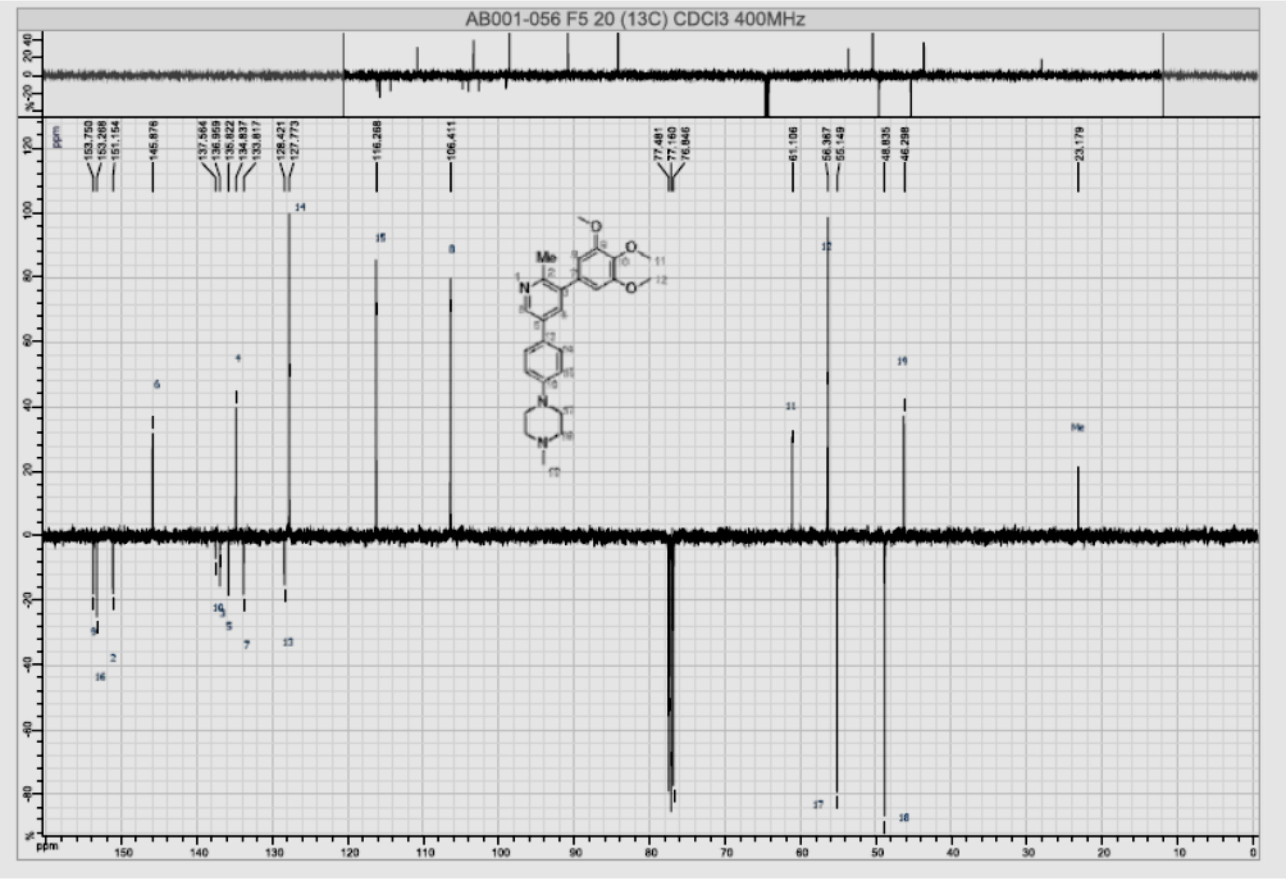
AB001-056 F5 20 (13C) CDCl3 400MHz
153.750
153.268
151.154
145.876
137.564
136.959
135.822
134.837
133.817
128.421
127.773
116.268
106.411
77.481
77.160
76.846
61.106
56.367
55.149
48.835
46.298
23.179
ppm
