# Supplementary material for: ALK2 inhibitors display beneficial effects in preclinical models of ACVR1 mutant diffuse intrinsic pontine glioma
Source: Commun Biol. 2019 May 9;2:156. doi: 10.1038/s42003-019-0420-8 (PMC6509210; doi:10.1038/s42003-019-0420-8)
Supplement: Supplementary file 3 — Description of Additional Supplementary Files [file 42003_2019_420_MOESM3_ESM.pdf]

Description of Additional Supplementary Files

File Name: Supplementary Data 1
Description: Gene expression differences in ACVR1 mutant versus wild-type 3 DIPG samples
