# Supplementary material for: ALK2 inhibitors display beneficial effects in preclinical models of ACVR1 mutant diffuse intrinsic pontine glioma
Source: Commun Biol. 2019 May 9;2:156. doi: 10.1038/s42003-019-0420-8 (PMC6509210; doi:10.1038/s42003-019-0420-8)
Supplement: Supplementary file 4 — Reporting Summary [file 42003_2019_420_MOESM4_ESM.pdf]

nature research

Corresponding author(s): Jones

Last updated by author(s): Mar 22, 2019

Reporting Summary

Nature Research wishes to improve the reproducibility of the work that we publish. This form provides structure for consistency and transparency in reporting. For further information on Nature Research policies, see Authors & Referees and the Editorial Policy Checklist.

Statistics

For all statistical analyses, confirm that the following items are present in the figure legend, table legend, main text, or Methods section.

n/a	Confirmed	
☐	☒	The exact sample size (*n*) for each experimental group/condition, given as a discrete number and unit of measurement
☐	☒	A statement on whether measurements were taken from distinct samples or whether the same sample was measured repeatedly
☐	☒	The statistical test(s) used AND whether they are one- or two-sided<br>*Only common tests should be described solely by name; describe more complex techniques in the Methods section.*
☐	☒	A description of all covariates tested
☐	☒	A description of any assumptions or corrections, such as tests of normality and adjustment for multiple comparisons
☐	☒	A full description of the statistical parameters including central tendency (e.g. means) or other basic estimates (e.g. regression coefficient) AND variation (e.g. standard deviation) or associated estimates of uncertainty (e.g. confidence intervals)
☐	☒	For null hypothesis testing, the test statistic (e.g. F, t, r) with confidence intervals, effect sizes, degrees of freedom and P value noted<br>*Give P values as exact values whenever suitable.*
☒	☐	For Bayesian analysis, information on the choice of priors and Markov chain Monte Carlo settings
☒	☐	For hierarchical and complex designs, identification of the appropriate level for tests and full reporting of outcomes
☒	☐	Estimates of effect sizes (e.g. Cohen's d, Pearson's r), indicating how they were calculated

Our web collection on statistics for biologists contains articles on many of the points above.

Software and code

Policy information about availability of computer code

Data collection	N/A
Data analysis	Gene Set Enrichment Analysis (software.broadinstitute.org/gsea) was performed using the GSEA java application

For manuscripts utilizing custom algorithms or software that are central to the research but not yet described in published literature, software must be made available to editors/reviewers. We strongly encourage code deposition in a community repository (e.g. GitHub). See the Nature Research guidelines for submitting code & software for further information.

Data

Policy information about availability of data

All manuscripts must include a data availability statement. This statement should provide the following information, where applicable:

- Accession codes, unique identifiers, or web links for publicly available datasets
- A list of figures that have associated raw data
- A description of any restrictions on data availability

The datasets generated during and/or analysed during the current study are available in the paediatric-specific implementation of the cBioPortal genomic data visualisation portal (pedcbioportal.org).

Field-specific reporting

Please select the one below that is the best fit for your research. If you are not sure, read the appropriate sections before making your selection.

☒ Life sciences ☐ Behavioural & social sciences ☐ Ecological, evolutionary & environmental sciences

Life sciences study design

All studies must disclose on these points even when the disclosure is negative.

Sample size	For in vivo efficacy experiments, we used standard sample sizes of up to n=10 per treatment arm, powered to detect a 10% increase in survival at an alpha=0.05, and power of 0.80.
Data exclusions	There were no data exclusions.
Replication	Interventional in vitro experiments were carried out with at least n-=3 biological replicates.
Randomization	Animals were randomised to each treatment arm.
Blinding	Biomarker analyses on mouse tissue was carried out and analysed blinded to the treatment given.

Reporting for specific materials, systems and methods

We require information from authors about some types of materials, experimental systems and methods used in many studies. Here, indicate whether each material, system or method listed is relevant to your study. If you are not sure if a list item applies to your research, read the appropriate section before selecting a response.

Materials & experimental systems

n/a	Involved in the study	
☐	☒	Antibodies
☐	☒	Eukaryotic cell lines
☒	☐	Palaeontology
☐	☒	Animals and other organisms
☒	☐	Human research participants
☒	☐	Clinical data

Methods

n/a	Involved in the study	
☒	☐	ChIP-seq
☒	☐	Flow cytometry
☒	☐	MRI-based neuroimaging

Antibodies

Antibodies used	Pressure antigen retrieval was performed and staining was carried out using antibodies directed against Ki67 (DAKO, #7240, 1:100), and CD31 (Abcam, #28364, 1:50).
Validation	Validated using appropriate positive control tissue as recommended by the manufacturer, as well as no primary antibody negative controls.

Eukaryotic cell lines

Policy information about cell lines

Cell line source(s)	Patient-derived primary cultures developed by the authors.
Authentication	STR profiling, custom panel and exome sequencing.
Mycoplasma contamination	All were verified mycoplasma free.
Commonly misidentified lines (See ICLAC register)	No commonly misidentified lines were used

Animals and other organisms

Policy information about studies involving animals; ARRIVE guidelines recommended for reporting animal research

Laboratory animals	BALB/c and NOD-SCID mice, female, aged 3-6 weeks.
Wild animals	The study did not involve wild animals
Field-collected samples	The study did not include field-collected samples
Ethics oversight	Institute of Cancer Research AWERB, Comite Etico de Experimentacion Animal at Universidad de Barcelona

Note that full information on the approval of the study protocol must also be provided in the manuscript.
